# Awake rodent fMRI: Gradient-echo echo planar imaging versus compressed-sensing fast low-angle shot

**DOI:** 10.1162/imag_a_00406

**Published:** 2025-01-02

**Authors:** Christopher Cover, Sujatha Reddy, Alberto Vazquez, Mitsuhiro Fukuda, Alexander J. Poplawsky

**Affiliations:** Department of Radiology, University of Pittsburgh, McGowan Institute for Regenerative Medicine Building, Pittsburgh, PA, United States; Department of Bioengineering, University of Pittsburgh, Pittsburgh, PA, United States

**Keywords:** awake rodent fMRI, olfactory bulb, high-resolution fMRI, CBVw fMRI, functional pulse sequence comparison

## Abstract

Awake rodent functional magnetic resonance imaging (fMRI) is increasingly becoming a reliable neuroimaging technique to study neuronal activity at both the whole-brain and high-resolution laminar scales. Prior studies have focused on developing acclimation protocols, experimental paradigms, and hardware to optimize outcomes. However, little effort has been made to address the impact of pulse sequence selection on detecting brain activation in awake fMRI experiments. In the current study, we compare gradient-echo echo planar imaging (GE-EPI) and compressed-sensing fast low-angle shot (CS-FLASH) sequences with cerebral blood volume-weighted (CBVw) contrast enhancement to investigate their sensitivity to hemodynamic activity in the olfactory bulb of awake rodents. Compared with GE-EPI, CS-FLASH had comparable motion parameters but was more sensitive to large motions, often resulting in corruption of the image quality. The use of framewise displacement as a motion censoring technique may over censor the data, requiring alternative approaches, such as spatial correlation censoring. CS-FLASH images were qualitatively sharper than GE-EPI; however, the contrast-to-noise ratio for odor activation was consistently greater for GE-EPI than for CS-FLASH that cannot be explained by olfactory adaptation alone. The activation maps of CS-FLASH to four different odors showed spatially unique patterns consistent with GE-EPI, but with lower z-scores or detection sensitivity. Activation maps were consistent with previously established histological findings. Additionally, odor-evoked laminar activation was greatest in the superficial layers that decreased with laminar depth, consistent with prior findings. We conclude that CS-FLASH produces sharper images with equivalent spatial activation maps to GE-EPI, albeit with lower statistical strength and contrast-to-noise ratio (CNR), and without being prohibited by motion-related image distortion.

## 1 Introduction

Awake rodent functional magnetic resonance imaging (fMRI) has emerged as a reliable neuroimaging technique to study the brain during task (Cover et al., 2021; Han et al., 2019; Sakurai et al., 2020), passive stimulus (Chen et al., 2020; Desai et al., 2011; Dinh et al., 2021; Liang et al., 2015), resting-state conditions (Becerra et al., 2011; Gutierrez-Barragan et al., 2022; Zhang et al., 2010), and at high spatial resolutions (Poplawsky et al., 2023). The elimination of anesthetics to reduce motion contamination of the functional data improves the sensitivity of the hemodynamic response, allowing for more robust interpretation of the neurovascular data (Desai et al., 2011; Dinh et al., 2021). Much effort has gone into experimental design (Chen et al., 2020; Cover et al., 2021), rodent acclimation and restraint (Harris et al., 2015; King et al., 2005; Tsurugizawa et al., 2020; Yoshida et al., 2016), and data preprocessing (Chen et al., 2020; Dinh et al., 2021; Gutierrez-Barragan et al., 2022) to ensure that the outcome data have minimal contamination of motion and animal stress. Though previous efforts have been instrumental in making awake rodent fMRI a viable neuroimaging technique, they have predominately utilized traditional gradient-echo echo planar imaging (GE-EPI) to study neurovascular activity. To date, no study has compared the impact of pulse sequence selection on functional activation metrics in awake fMRI of rodents.

While GE-EPI is the standard pulse sequence for fMRI studies due to its rapid acquisition of data (0.5–2 s temporal resolution) and sensitivity to endogenous changes in hemodynamic activity (e.g., blood oxygenation level dependent, BOLD, signal), it is particularly vulnerable to geometric distortions and signal dropout at boundaries with large magnetic susceptibility differences (e.g., air–tissue interfaces). This loss of signal may preclude reliable imaging in cortical areas such as near the aural cavity or in the olfactory bulb (OB) in rodents. Additionally, due to T2* decay, acquisition of k-space following a single excitation pulse results in low available signal in k-space regions carrying high spatial frequencies, limiting image resolution. Fast low-angle shot (FLASH) has been used as an alternative pulse sequence to measure hemodynamic activity in areas of high signal dropout (Kida et al., 2002; Poplawsky & Kim, 2014; Poplawsky et al., 2019b; Xu et al., 2000; Zong et al., 2014). Compared with GE-EPI, FLASH acquires a single line of k-space per excitation pulse that limits T_2_* decay and improves image sharpness at high resolutions, but at the cost of poor temporal resolution. To improve the temporal resolution, compressed-sensing (CS) techniques have been applied to FLASH (i.e., CS-FLASH). CS-FLASH improves the temporal resolution by sampling less k-space and taking advantage of temporal redundancies in k-space to estimate lines that were not acquired (Zong et al., 2014). This approach effectively captures high spatial frequency data at accelerated speeds, although still slower than GE-EPI. Both FLASH and CS-FLASH have been utilized as alternative functional imaging techniques for high-resolution imaging in areas with high magnetic susceptibilities in anesthetized animals. However, it is currently unknown how well a high-fidelity imaging technique, like CS-FLASH, will perform at high spatial resolutions in the awake condition with increased motion.

Previously, our group established that high-resolution imaging of the OB using GE-EPI can reliably capture odor-specific activation maps of OB in awake mice with minimal motion contamination (Poplawsky et al., 2023). In the current study, we aim to address outstanding questions regarding the impact of pulse sequence selection on high-resolution imaging in awake mice. We compared GE-EPI and CS-FLASH pulse sequences and their sensitivity to hemodynamic cerebral blood volume-weighted (CBVw) activity in the olfactory bulb (OB) of awake mice. We compared previously published GE-EPI results (Poplawsky et al., 2023) with CS-FLASH data that were also collected during the same experiments. In short, CS-FLASH is a comparable imaging technique with GE-EPI for high-resolution imaging of the rodent OB. CS-FLASH produced similar motion characteristics to previously reported awake rodent imaging studies, and spatially unique activation maps that were consistent with prior literature, but had lower statistical strength and sensitivity than GE-EPI. Our results validate CS-FLASH as an alternative pulse sequence to GE-EPI to use in instances in which GE-EPI cannot provide high-quality images, especially in regions of high magnetic susceptibility, further expanding the toolset available to awake rodent imaging.

## 2 Methods

**Fig. 1.**
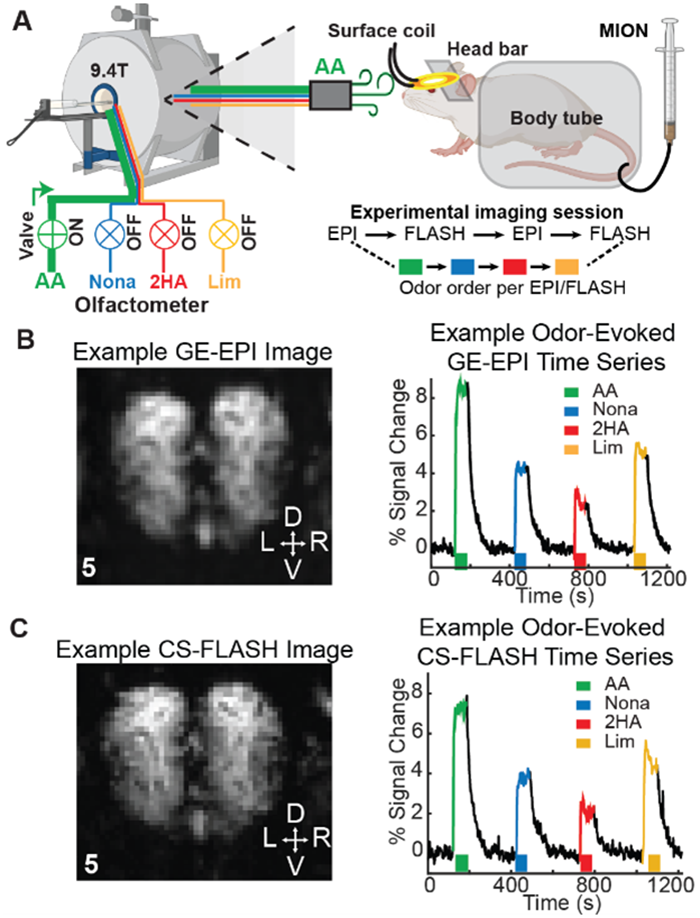
Overview of awake multi-pulse CBVw odor experiments. (A) Four odorants were delivered to mice (n = 7) in a 9.4-T Bruker scanner using a modified air-dilution olfactometer. Odorants were amyl acetate (AA; 50 mL odorized air + 950 mL blank air or 5% air dilution), nonanal (Nona; 15% air dilution), 2-hydroxyacetophenone (2HA; 10% air dilution), and limonene (Lim; 20% air dilution). The four odors were repeatedly presented in a sequential manner (AA → Nona → 2HA → Lim) in a box-car design (2-min blank air, 64-s odor delivery, 2-min blank air). Scan sessions consisted of alternating blocks of four odors with GE-EPI followed by four odors with CS-FLASH within the same scan day (GE-EPI → CS-FLASH → GE-EPI → CS-FLASH). Subpanel created at BioRender.com. Green arrow indicates airflow and direction. Colors and colored boxed indicates odor type; green—AA, blue—NA, red—2HA, and yellow—Lim. Example contrast-enhanced GE-EPI (B) and CS-FLASH (C) mean baseline images and time series within the olfactory bulb acquired during awake CBVw fMRI scans. The slice number is indicated in the lower left corner. D—dorsal, V—ventral, L—left, R—right.

All methods regarding animal preparation and surgery, MRI acclimation, MRI experimental set-up and experimental design, MRI acquisition and imaging, fMRI data processing, and analysis were previously described in detail (Poplawsky et al., 2023). Figure 1 provides a brief experimental overview and example functional images and time series. In short, all procedures, surgeries, and experiments followed the National Institutes of Health guide for the care and use of laboratory animals (NIH Publications No. 8023, revised 1978), and were approved by the University of Pittsburgh’s Institutional Animal Care and Use Committee. GE-EPI data in the following sections have been previously published in the following manuscript (Poplawsky et al., 2023). Any new analysis of GE-EPI data is described.

### 2.1 Animal preparation, surgery, and acclimation

Seven (n = 7) B6129SF1/J male mice (Jackson Laboratory, Bar Harbor, ME) underwent implantation of an acrylic headplate for head fixation during awake imaging. Mice were briefly exposed to 5% isoflurane for induction and then maintained at 1.5–2% for the length of the surgery. A lightweight, home-made acrylic headplate (~420 mg, 12 × 23 mm^2^, 1 mm thick, McMaster-Carr, Elmhurst, IL) was fixed with dental cement and Vetbond tissue adhesive to the exposed skull. Mice were provided antibiotics and analgesics for 3 days following the surgery and given 2 weeks to recover prior to handling and acclimation. To minimize motion and stress during fMRI scans, mice were acclimated to head fixation and body restraint (Ferenczi et al., 2016; King et al., 2005; Stenroos et al., 2018). Mice were acclimated (Supplementary Fig. 1B) every other day for 3 days a week, beginning with a 30-min session that was increased by 15 min per session until a maximum of 120 min was reached (i.e., 7 sessions). Starting at the 5^th^ session (i.e., 90-min acclimation), acoustic recordings of the GE-EPI and CS-FLASH fMRI sequences were introduced during the final 30 min of acclimation at 90 dB. The length of time and volume of the fMRI noise were systematically increased over the next five sessions until it was played for the entire 120 min at 120 dB (Miyati et al., 2001).

### 2.2 MRI compatible cradle and olfactometer

An MRI compatible cradle was designed using SketchUp (Supplementary Fig. 1C; Trimble, Westminster, CO) and 3D printed using a Ultimaker 2+ extended (Utrecht, Netherlands) with PLA plastic. The cradle consisted of four parts: (1) a Bruker rat cradle interface, (2) integrated Teflon tubing for delivering odors from the olfactometer to the mouse’s nose and vacuum port for odor removal, (3) plastic headplate holders, and (4) headplate locking clamps. All 3D-printed components can be downloaded at https://github.com/neuroimlabpitt/Olfactory-fMRI.

Odor was delivered via air dilution using a modified olfactometer (Knosys Olfactometers, Lutz, FL). Medical air continuously flowed through the olfactometer at 1 L/min. During odor exposure, air flow was diverted to one of four pure odorants at pre-set flow rates in the following order: amyl acetate (AA; 50 mL/min diverted or 5% air dilution), nonanal (Nona; 150 mL/min diverted or 15% air dilution), 2-hydroxyacetophenone (2HA; 100 mL/min diverted or 10% air dilution), and (+)-limonene (Lim; 200 mL/min diverted or 20% air dilution). AA was chosen first since it robustly activates the bulb, and was used as a positive control to quickly ensure proper odor delivery. The odorant order was fixed to mitigate olfactory adaptation by maximizing the time between exposures to the same odorant (i.e., ~20 min). Check valves were placed at the end of each odor line where they converged into a single tube, ~30 cm from the mouse nose, to prevent backflow and residual odor mixing. In addition, a vacuum line ~1 cm from the mouse nose and 4 min of blank air between odorants aided in isolation of odorant presentation. Odor concentrations were selected in a preliminary study based on whether a single odor trial could reliably produce robust bulb activations (data not shown).

### 2.3 General MRI acquisition and procedures

Each mouse underwent a total of 2–3 imaging sessions in this study. MRI data were acquired using a 9.4 T/30-cm AVIII HD spectrometer (Bruker Biospin, Billerica, MA) with a 12-cm high-performance gradient set and ParaVision 6.0.1. An 86-mm quadrature volume coil was used for excitation, while a 10-mm surface coil centered over the olfactory bulb was used for signal reception. Prior to scans, the mouse tail vein was injected with monocrystalline iron oxide nanoparticles (MION; 25 mg Fe/kg, Fereheme, AMAG Pharmaceuticals, MA) under 1.5% isoflurane (<15-min duration) for contrast-enhanced CBVw fMRI (Poplawsky et al., 2019a). Mice were then head fixed in the MRI compatible cradle and allowed to recover for 30–45 min before fMRI acquisition. To reduce field inhomogeneities from the nasal sinus, shimming was localized to the OB only.

### 2.4 Anatomical imaging

We identified the anterior commissure using a T2-weighted anatomical RARE sequence (2 s TR, 13.6 ms TE, 2 averages, RARE factor of 4, 11 × 11 mm^2^ field-of-view (FOV), 128 × 128 matrix size, 21 slices) to standardize the fMRI slice alignment in the olfactory bulb across the fMRI imaging sessions. The center slice of the fMRI volume was then positioned 4.15–4.25 mm anterior to AC into OB.

### 2.5 fMRI data acquisition

GE-EPI imaging parameters (Table 1) were as follows: 2 segments, 1 s TR (i.e., 2-s effective TR to match CS-FLASH), 7.5 ms TE, 65º Ernst flip angle, 6.4 × 6.4 mm^2^ FOV, 64 × 64 matrix (i.e., 100 × 100 µm^2^ in-plane voxel size), 9 slices, 300-µm slice thickness, 178,571.4 kHz sampling bandwidth, no spatial saturation, partial FT encoding to achieve an effective acceleration of 1.39, and the default navigator pulse with automatic ghost correction. An inter-volume delay of 806.48 ms captured the entire volume within 193.52 ms of the TR to reduce the impact of motion artifacts on imaging data. CS-FLASH imaging parameters (Table 1) were as follows: 125 ms TR (2 s effective TR), 7.5 ms TE, 35º Ernst flip angle, 6.4 × 6.4 mm^2^ FOV, 64 × 16 matrix (64 × 64 reconstructed matrix, reduction factor of 4, 100 × 100 µm^2^ in-plane voxel size), 9 slices, 300-µm slice thickness, 10 kHz sampling bandwidth, 60 dummy scans, and no spatial saturation. K-space was acquired in an optimal RAND + C6 pattern, as previously determined (Zong et al., 2014), which is constantly sampling the central 6 lines of k-space for each slice in addition to a random sampling of 10 other lines. All lines were sampled in numerical order throughout k-space acquisition. Random acquisition of lines outside of the central six ensures appropriate sampling of the edges of k-space for high spatial resolution imaging. A single fMRI run was 152 TRs (i.e., 304 s or ~5 min) and consisted of a 60 TR baseline (120 s), 32 TR odor exposure (64 s), and 60 TR post-stimulus period (120 s). The pulse sequence alternated between GE-EPI and CS-FLASH (4 odors GE-EPI →4 odors CS-FLASH→4 odors GE-EPI → 4 odors CS-FLASH), for a total of 16 fMRI runs per scan session. Each sequence cycle consisted of a fixed presentation of four odors (AA→ Nona → 2HA → Lim).

**Table 1.** Pulse sequence parameters for GE-EPI versus CS-FLASH.

	GE-EPI	CS-FLASH
Repetition time (TR)	1 s	125 ms
Effective TR	2 s	2 s
Echo time (TE)	7.5 ms	7.5 ms
Ernst flip angle	65º	35º
Field-of-view (FOV)	6.4 × 6.4 mm^2^	6.4 × 6.4 mm^2^
Matrix	64 × 64	64 × 16
Number of slices	9 (300-µm thickness)	9 (300-µm thickness)
Sampling bandwidth	178,571.4 kHz	10 kHz
K-space sampling	Zig-zag traversal	Rand + C6

### 2.6 fMRI data processing and analysis

All data were processed using the Analysis of Functional NeuroImages (AFNI) software (Cox, 1996; Cox & Hyde, 1997; Gold et al., 1998) and MATLAB (version 2019b, Natick, MA). The following analysis pipeline was applied to both GE-EPI and CS-FLASH data.

#### 2.6.1 Individual and group analysis

All individual fMRI runs from a scan session were temporally concatenated and motion corrected to the first fMRI volume with *3dvolreg* in AFNI. Motion parameter estimates (Δx, Δy, Δz, roll, pitch, yaw) were used to calculate each volume’s Euclidean framewise displacement (FD) for motion censoring purposes (Cover et al., 2021; Han et al., 2019; Power et al., 2014). Volumes with FD values exceeding 25 µm, or ¼ of a voxel, were excluded from further analysis, as in our previous study. AFNI’s *3dREMLfit* was used to calculate β-estimates and t-maps using a design matrix containing predictor variables for all four odors using impulse response functions based on a previous CBVw study (Silva et al., 2007), in addition to the six motion parameter estimates, linear drift, and baseline constant covariates.

Baseline fMRI images from each session were normalized to a representative baseline fMRI template scan from our study using *3dAllineate* with an lpa cost function and *3dQwarp*, and linearly interpolated to a higher in-plane resolution (128 × 128 matrix size, 50 × 50 µm^2^). These transformational values for each session were then applied to the individual volumes of the fMRI runs, for laminar time-series analysis; as well as the odor-evoked β-estimates and t-maps, for group activation maps. Group z-maps with FDR correction were calculated from normalized β- and t-maps with *3dMEMA* and *3dFDR.* Z-score thresholds of the top 1%, 2.5%, 5%, and 10% were used to control for variations in activation strength and determine spatial overlap of odor activations. All maps had a minimum voxel cluster of 10.

#### 2.6.2 Flat map generation and center-of-mass calculation

To visualize three-dimensional odor-evoked activity in two dimensions, we created flat maps of the glomerular layer (GL) (Poplawsky et al., 2023; Sanganahalli et al., 2020; Xu et al., 2003). Since GE-EPI and CS-FLASH data were normalized to the same image space as our previous manuscript, we utilized the same laminar masks (see section 2.8.3 in Poplawsky et al., 2023). Flat maps were calculated along GL only (200-µm thick or two native fMRI voxels) from individual t-maps. To linearize GL, we used a moving average 250 × 250 µm^2^ square that started at the ventral-most point and progressed circularly in the lateral direction to obtain the mean t-value for that point. Only voxels within GL were included in the analysis (Fig. 4A). This flattening process was repeated for each slice and hemi-bulb to create a whole-bulb flat map of GL. Flat maps for the left and right bulbs were averaged due to their qualitative similarity, followed by a group averaging.

To further quantify whether the flat map activations were unique to the odor, we calculated the weighted center of masses (CoM). Consistent with group activation maps, we only looked at the top 5% of flat map t-values for CoM calculations. Qualitative analysis of flat maps revealed two distinct regions of activation; we, therefore, drew ROIs around these distinct clusters of activation in the ventromedial and dorsolateral regions of the OB for each odor (Fig. 4B). The CoM for each ROI was then calculated. To estimate the reproducibility of activation patterns, we calculated the Euclidean distance of CoM values between different scan days for each mouse. For mice that were scanned more than twice, we used the centermost CoM value as the reference point.

#### 2.6.3 Layer-dependent analysis

The mean signal change from all voxels in each layer was calculated before averaging them across imaging sessions. For relative laminar changes between odors, these values were further normalized by the mean signal change of all six layers (Fig. 5A).

#### 2.6.4 Laminar time-series and contrast-to-noise ratio analyses

On a voxel-wise basis, we linearly detrended the time series and calculated the percent signal change (ΔS/S_0_) for each odor run using 0–120 s and 280–304 s as our baseline time periods. We then averaged the two repeated runs for each of the four odors before finally averaging across the fMRI sessions. Missing values in time due to motion censoring were ignored and the number of sessions for each time point used in the Standard Error of the Mean (SEM) calculation was adjusted accordingly. We further calculated the contrast-to-noise ratio (CNR) by dividing the mean odor-evoked signal change during peak activation by the standard deviation of the pre-stimulus baseline (0–120 s) for all voxels in GL (i.e., no threshold). The temporal range for the peak activation was previously determined when the mean signal change in the whole bulb, independent of layer, maintained 80% of its maximum (Fig. 5B, vertical dashed lines), which were determined in the GE-EPI time series and applied to CS-FLASH data (Poplawsky et al., 2023).

### 2.7 Statistical analysis

Statistical analyses were run in GraphPad Prism Version 9.4.1 or MATLAB Version 2019b for univariate analysis and IBM SPSS Version 28.0.1.1 for multivariate analysis. Normality for all datasets were tested using a Kolmogrov–Smirnov test to determine whether parametric or non-parametric techniques should be used. For univariate parametric techniques, we used a one- or two-way ANOVA with a Geisser–Greenhouse sphericity correction and Šidák’s post hoc tests corrected for multiple comparisons. A paired t-test was used for simple pairwise comparisons since GE-EPI and CS-FLASH were acquired within the same mouse on the same scan day. Data points greater than 3 standard deviations from the mean were considered outliers and the associated dataset was assessed for quality integrity. Outliers from datasets that passed quality assessment were not discarded from analysis but identified within figures and text of this publication. For multivariate parametric analyses, we used a one-way MANOVA with Šidák’s post hoc tests for multiple comparisons. For non-parametric tests, we used a Kruskal–Wallis test with Šidák’s post hoc test for multiple comparisons, and a Wilcoxon Rank sum with corrected p-value for post hoc analysis. To calculate t- and p-values from Pearson’s correlation coefficients, r, we used a Fisher transformation. All data are expressed as mean ± SEM, unless otherwise noted.

## 3 Results

### 3.1 Motion characterization of awake CS-FLASH scans and comparison with GE-EPI

Imaging awake mice increases the likelihood of motion contamination of the functional data. CS-FLASH is more sensitive to image corruption due to motion because of its slower acquisition speed compared with GE-EPI (Supplementary Figs. 2 and 3 for motion and image quality in CS-FLASH and GE-EPI, respectively). Overall, the motion characteristics of the awake CS-FLASH data were comparable with our prior GE-EPI publication and previously reported observations in the literature (Chen et al., 2020; Cover et al., 2021; Han et al., 2019; Poplawsky et al., 2023; Tsurugizawa et al., 2020). Similar to the GE-EPI data, the motion estimates of CS-FLASH data had a gamma distribution, so median and median absolute deviation (MAD) were used to report the data.

**Fig. 2.**
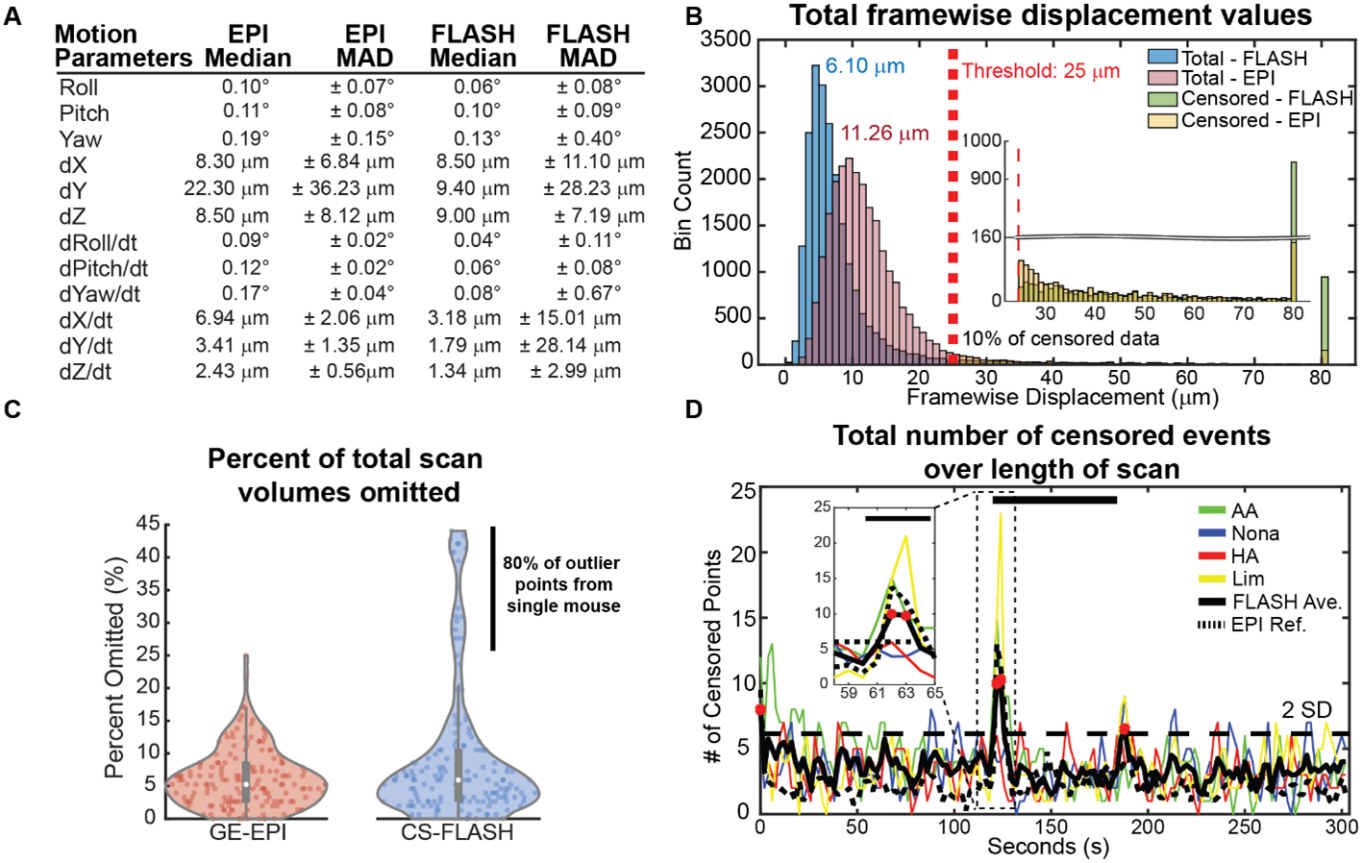
Comparative motion characteristics of awake mice during olfactory fMRI scans, GE-EPI versus CS-FLASH. (A) AFNI’s *3dVolReg* motion parameter estimates of 5-min runs broken down into their median and median absolute deviation (MAD) rigid body components and first derivative components. (B) Histogram of framewise displacement (FD) values for all GE-EPI (i.e., EPI) and CS-FLASH (i.e., FLASH) volumes. Median values following censoring are 11.26 µm and 6.10 µm for GE-EPI and CS-FLASH, respectively. (C) Total percent of TR’s censored per GE-EPI or CS-FLASH scan. For GE-EPI, 5.26% ± 4.4% (8 ± 7 TR’s out of 152) was censored. For CS-FLASH, 4.61 ± 9.51% (7 ± 14 TR’s out of 152) was censored. (D) Total number of censored events in time for each ~5-min run. Red dots indicate time points 2 SD above mean and correspond to the scan initiation (1^st^ TR), odor onset, and odor offset. The maximum possible number of censored points per time point is 38.

The motion parameter estimates (median ± MAD) for all awake 5-min CS-FLASH runs were 0.06º ± 0.08º roll, 0.10º ± 0.09º pitch, 0.13º ± 0.40º yaw, 8.50 ± 11.10 µm dX (left-right axis), 9.40 ± 28.23 µm dY (dorsal-ventral axis), and 9.00 ± 7.19 µm dZ (rostral-caudal axis) (Fig. 2A). Motion estimates were not statistically different between odors for CS-FLASH (Supplementary Table 1, Kruskal–Wallis Test, Bonferroni correct p-value for multiple comparisons with p < 0.0125 considered significant) and was only statistically different between pulse sequences (i.e., CS-FLASH vs. GE-EPI) for motion in the yaw rotation (Supplementary Table 2, Kruskal–Wallis Test, Bonferroni correct p-value for multiple comparisons with p < 0.0125 considered significant). Median values were similar for two of the three translational planes for CS-FLASH but differed in relative motion observed in the dorsal-ventral axis (dY: 9.40 vs. 22.30 µm, CS-FLASH and GE-EPI, respectively). In our prior work, we qualitatively determined that the large median dY displacement value for GE-EPI was due to a mixture of slow drifts and sustained shifts in head position, but not sporadic motion throughout time (Poplawsky et al., 2023). Sporadic motion was estimated by looking at the change in motion over time (i.e., dY/dt). Interestingly, while the motion derivate reduced the CS-FLASH median dY motion contribution by 81% (dY 9.40 µm → dY/dt 1.79 µm), its MAD only reduced by 0.4% (dY 28.23 µm → dY/dt 28.14 µm). This suggests that sporadic motion explains a lot of the variance within the CS-FLASH data, in comparison with GE-EPI. Qualitative analysis of the dY motion parameter time series revealed that 2% of scans had a sudden and sustained shift in head position, 11% had a slow drift in head position, 0% had mixed shift and drift, and 88% had sporadic change that quickly returned to baseline levels of motion.

FD is used in awake rodent and human resting-state imaging to identify and omit volumes with motion exceeding a pre-established threshold (Chen et al., 2020; Cover et al., 2021; Han et al., 2019; Power et al., 2014), which is 25 µm, or ¼ of an in-plane voxel, in the current study. FD is calculated as the Euclidean distance between two successive scan volumes and is, therefore, a first-derivate scalar estimate of motion. Overall, the median FD value for CS-FLASH scans was 6.50 ± 58.7 µm (median ± MAD) before censoring and 6.10 ± 2.9 µm after censoring. This represents a 6% reduction in the median amount of motion, but a 95% reduction in the median absolute deviation (Fig. 2B). FD values were not dependent on odor type (Kruskal–Wallis Test before censoring, χ^2^(159) = 1.08, p = 0.78; one-way ANOVA after censoring, F(159) = 0.76, p = 0.519).

When comparing FD distributions between the pulse sequences, two distinct features were observed (Fig. 2B). First, the number of volumes with FD exceeding 80 µm was ~6 times greater for CS-FLASH compared with GE-EPI. Since CS-FLASH is more sensitive to image corruption due to motion because of its slower acquisition speed compared with GE-EPI (Supplementary Figs. 2 and 3 for motion and image quality in CS-FLASH and GE-EPI, respectively), we examined whether corrupted images produced large FD values. Indeed, volumes with poor spatial correlation (r < 0.95) with the mean image in time (i.e., corrupt images) had higher FD values than volumes with low image distortion, FD_high_ 59.9 ± 25.59 µm versus FD_low_ 6.1 ± 2.8 µm (median ± MAD; Wilcoxon Rank Sum p < 0.001). Second, the distribution of FD values was significantly less for CS-FLASH compared with GE-EPI after censoring; 6.10 ± 2.88 µm versus 11.26 ± 3.53 µm, respectively (Wilcoxon Rank Sum, p < 0.001). Interestingly, we found that image volumes with good spatial correlation (r_spatial_ > 0.95) that followed volumes with high image corruption also had high FD values (407 ± 452 µm vs. 438 ± 472 µm, respectively; Wilcoxon Rank Sum, p = 0.302), and are, subsequently, censored due to the 1^st^ derivative nature of FD. Together, CS-FLASH seems to be less sensitive to smaller motions but more sensitive to larger motions in an “all-or-nothing” manner.

Similar to GE-EPI data, an FD censoring threshold of 25 µm resulted in removal of 4.61 ± 9.51% (median ± MAD) or 7 ± 14 out 152 volumes per CS-FLASH run. In total, 9.74% of CS-FLASH data were omitted and 9.52% of GE-EPI data were omitted. The median number of omitted volumes per run was not dependent on odorant (Kruskal–Wallis Test, χ^2^(166) = 3.83, p = 0.28). However, compared with GE-EPI data, CS-FLASH had more individual runs with a larger average number of omitted volumes (Fig. 2C, black vertical bar). We sought to determine whether these runs were scattered across the total cohort of mice or belonged to specific mice. We found that most runs (24/30, i.e., 80%) with an omitted volume greater than 20% belonged to a single mouse (Supplementary Fig. 4C). Next, we investigated when motion occurred over the length of the 5-min run (Fig. 2D; dotted black line is average GE-EPI censoring for reference). Like GE-EPI, time points were most likely censored at the beginning of the run (1 volume, 2 s) or following odor onset (2 volumes, 4 s). Interestingly, we saw a third instance of increased motion during CS-FLASH runs, following cessation of the odor (1 volume, 2 s). Similar to GE-EPI, we saw that AA and Lim evoked more censored time points during the odor onset than 2HA and Nona. Unlike GE-EPI data, Lim odor was associated with more censored volumes during odor onset than AA for CS-FLASH data. Lastly, we wanted to determine whether motion censoring increased with time in the scanner. Unlike GE-EPI, we did find a significant negative correlation between time in the scanner and number of censored volumes in our CS-FLASH data (Pearson’s r = -0.19, p = 0.01; Supplementary Fig. 4A). Given that this relationship did not exist in our GE-EPI data (Pearson’s r = 0.03, p = 0.68), we investigated whether this was related to the outlier mouse with higher-than-normal motion parameters. After omitting data from this mouse, we observed no relationship between time in the scanner and number of censored volumes (Pearson’s r = 0.09, p = 0.28; Supplementary Fig. 4B). It is worth noting that CS-FLASH was consistently at the end of the sequence cycle, with mice being in the scanner ~20 min longer than the last GE-EPI run.

Lastly, we examined whether there was a correlation between body motion and censored time points. To non-invasively monitor body motion, we utilized a pneumatic pressure sensor placed between the mouse’s back and the body tube for real-time recording (Supplementary Figs. 2 and 3, termed “Body Motion”). Since body motion occurred randomly throughout the length of a scan, a threshold-based detection was used to determine when large body motions events. An event was defined as a pneumatic signal change that was 2-times greater than the standard deviation of the signal. The 2 standard deviation threshold was manually quality checked to confirm it was a sensitive and specific threshold. The median number of body motion events was 6 ± 4.3 per CS-FLASH scan, which did not differ from GE-EPI (Wilcoxon Rank Sum, p = 0.24). For each 5-min run, 39 ± 27% of censored volumes coincided with body motion, while 34 ± 27% did not. Additionally, 26 ± 27% of body motion events did not lead to censoring. These values were not dependent on odor type (Kruskal–Wallis Test for simultaneous censored volumes and body motion: χ^2^(166) = 4.94, p = 0.18; Kruskal–Wallis Test for censored volumes without body motion: χ^2^(166) = 1.57, p = 0.67; and Kruskal–Wallis Test for body motion without volume being censored: χ^2^(166) = 3.37, p = 0.34). These values were consistent with our previously published GE-EPI data in which ~1/3 of censored volumes coincided with body motion, while ~1/3 of body motion events did not lead to a censored volume.

### 3.2 Odor-evoked fMRI activation maps: CS-FLASH versus GE-EPI

**Fig. 3.**
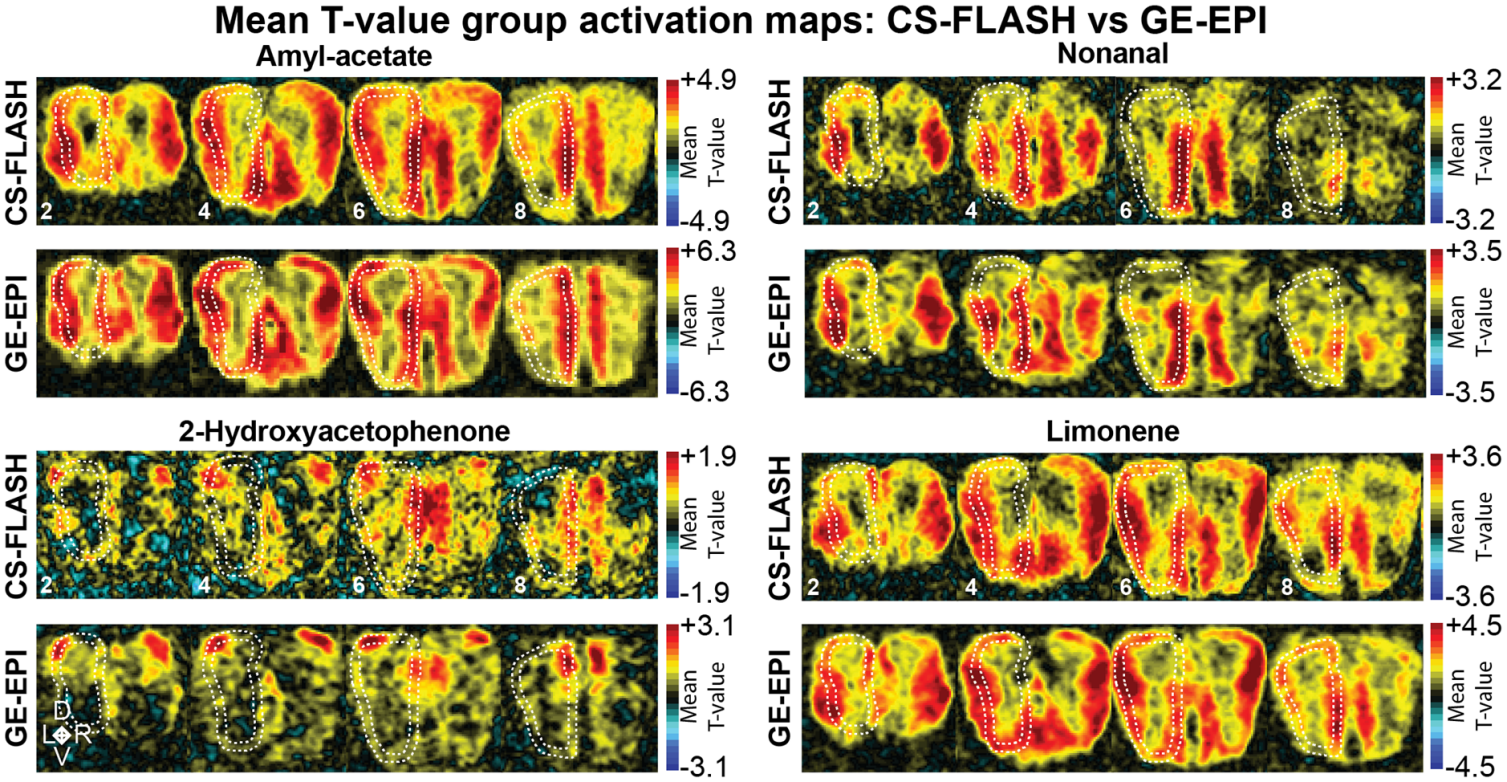
Mean t-value group activation maps: CS-FLASH versus GE-EPI. T-maps generated from *3dREMLfit* were normalized and averaged (n = 19 sessions, 7 mice, 2–3 sessions/mouse) for each odorant and are displayed without a threshold applied. Rostral OB slices are indicated with a lower slice number (see Amyl acetate panels) and progress caudally. The input layer, GL, is outlined with dotted white lines. Mean t-maps are for contrast purposes only and are not intended for statistical inference.

The glomerular organization within the rodent olfactory bulb produces discrete spatial activation patterns that are specific to the odorant. In our prior publication, we found that high-resolution (100 × 100 × 300 µm^3^) CBVw GE-EPI could resolve these unique spatial patterns of activity to four different odors (see Supplementary Figs. 5–7 for direct comparisons of GE-EPI and CS-FLASH mean t-value maps, top 5% activation maps, and odorant spatial overlap maps). In the current study, we are comparing whether CS-FLASH activation patterns could also measure similar discrete activation maps as GE-EPI. As before, two pairs of odors were chosen for having spatially distinct (2HA, Nona) and spatially similar (AA, Lim) activation maps. Mean t-value group maps (Fig. 3; Supplementary Fig. 5 for all slices) without a threshold applied showed similar spatial distributions of activity throughout the bulb when comparing pulse sequences of the same odorants. The spatial pattern also highlights the unique difference in pulse sequences. Compared with CS-FLASH, the spatial profile of GE-EPI values seems qualitatively “smoothed.” Mean t-value maps and top 5% CS-FLASH activation maps (Supplementary Fig. 6 for comparison with GE-EPI) were comparable with odor-evoked 2-deoxyglucose (2DG) maps previously reported in the literature for AA and Lim (Johnson et al., 1998, 2002, 2009), Nona (Johnson et al., 2009), and 2HA (Smear et al., 2013). AA and Lim predominately activated lateral and ventromedial regions of the OB, 2HA activated dorsolateral and dorsomedial regions, and Nona activated ventromedial and ventrolateral. To characterize the spatial separation of odors, we looked at the overlap (∩) of active voxels at top 10%, 5%, 2.5%, and 1% thresholds (Supplementary Fig. 7). As anticipated, increasing the threshold increased the spatial specificity of all four odors by increasing the proportion of voxels activated by a single odor: 56.6% (top 10% threshold), 70.1% (top 5%), 83.2% (top 2.5%), and 93.1% (top 1%). For the top 5% of active voxels, 2HA and Nona, and 2HA and Lim had highly discrete activation patterns with very little overlap of 1.3% and 1.4%, respectively. In comparison, AA and Lim had the largest degree of spatial overlap at 4.9%, like GE-EPI (Supplementary Fig. 7). A spatial overlap of three odors accounted for 6.4% of total active voxels, while an overlap of all four odors accounted for 0.4%.

**Fig. 4.**
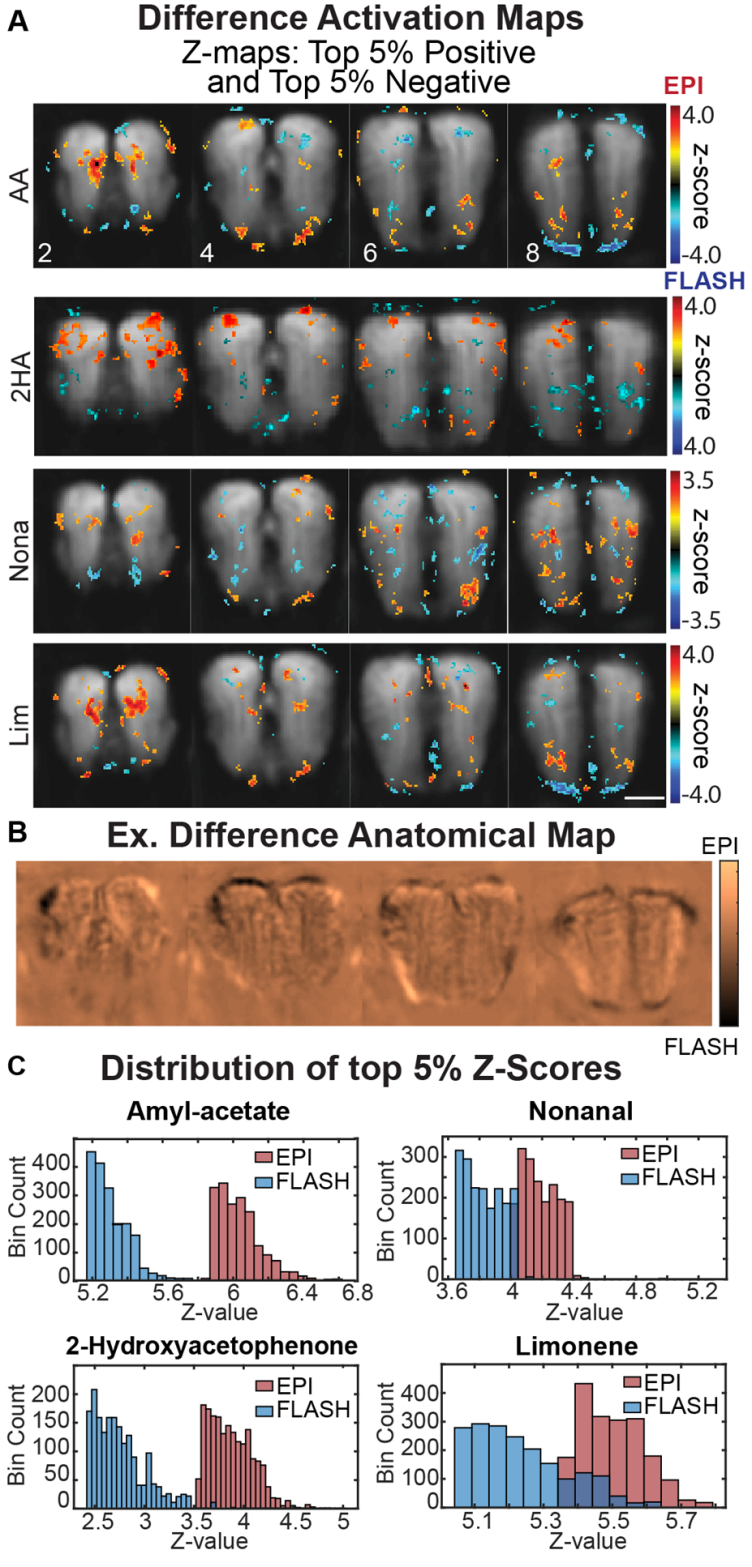
Group difference activation maps and comparison between CS-FLASH and GE-EPI. (A) Difference maps (GE-EPI–CS-FLASH) were generated to highlight differences in activation patterns. Top and bottom 5% of the difference maps are presented to show GE-EPI (warm colors) versus CS-FLASH (cold colors) predominance. (B) Difference baseline fMRI maps (min-max normalized, GE-EPI–CS-FLASH) were generated to highlight anatomical differences with increased signal for GE-EPI (brighter colors) versus CS-FLASH (darker colors). (C) Histogram distribution of top 5% of voxel z-scores per odor. AA—amyl acetate, Nona—nonanal, 2HA—2-hydroxyacetophenone, Lim—limonene.

Despite the general similarity of the GE-EPI and CS-FLASH odor maps to 2DG maps, qualitatively, GE-EPI and CS-FLASH provided complementary activation patterns. Additionally, CS-FLASH provided sharper images than GE-EPI (Fig. 1B, C). To contrast the pulse sequences, we calculated the difference between the two group activation maps and plotted the top 5% positive and top 5% negative difference values (Fig. 4A). Interestingly, CS-FLASH had a larger activation of the ventrocaudal bulb compared with GE-EPI (Fig. 4A, cold colors, slice 8 in AA and Lim). To investigate this further, the group baseline images after spatial normalization were compared for each sequence. These baseline images were first normalized to have the same dynamic range, then CS-FLASH was subtracted from GE-EPI. The resulting image showed larger baseline signal in the ventrocaudal bulb for CS-FLASH (Fig. 4B, dark region) than for GE-EPI, suggesting better sensitivity here for the former. On the contrary, GE-EPI had stronger activation in some bulb areas (Fig. 4A, warm colors), which may indicate a general increase in detection sensitivity compared with CS-FLASH. To examine this, we plotted the top 5% z-score distribution and found that CS-FLASH had a lower group map dynamic range in than GE-EPI with no or small degrees of overlap (Fig. 4C). This higher sensitivity to detecting odor-evoked changes may be attributed to the longer TR for GE-EPI (1,000 ms) than for CS-FLASH (125 ms).

In the previous section, we noted that utilizing FD to censor high motion volumes may be over censoring the CS-FLASH data in comparison with GE-EPI. We investigated whether censoring using spatial correlation alone would be a better tool to identify high-motion volumes while limiting over censoring in CS-FLASH data (Supplementary Fig. 8, top 5% threshold). Indeed, censoring using a spatial correlation (censored if r_spatial_ < 0.95) led to a 44% reduction in the number of censored volumes. However, activation maps that used spatial correlation censoring provided very comparable spatial activation patterns for all four odors with comparable dynamic range of z-scores as FD censored maps.

### 3.3 Odor-evoked flat maps: CS-FLASH versus GE-EPI

**Fig. 5.**
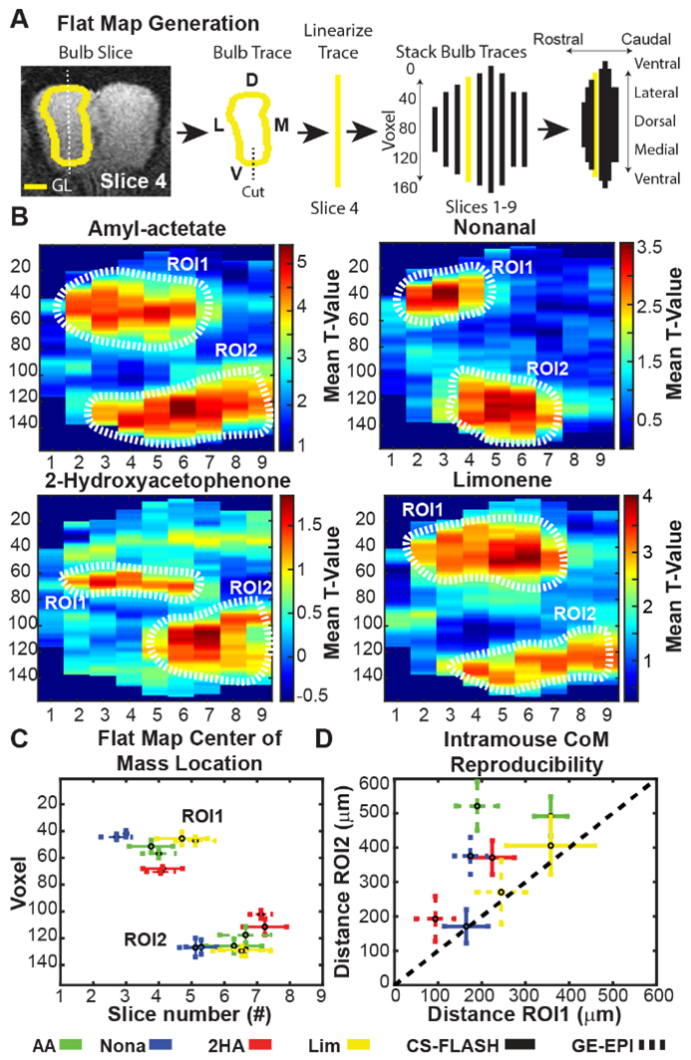
Odorant flat maps of the olfactory bulb with CS-FLASH versus GE-EPI. (A) Outline of flat map generation from the glomerular layer (GL, yellow line) of the olfactory bulb and orientation key of flat maps (far right). (B) Flat map representation of each odor. Vertical scale represents in-plane voxel number (50 µm/voxel), while horizontal scale represents slice number (300 µm/slice). Individual session t-maps (n = 17) were flattened and maps from both hemispheres were averaged (n = 34). Two regions of interests (ROI) were identified and outlined (dotted white line) in the dorsolateral and ventromedial bulb regions, ROI1 and ROI2, respectively. (C) Weighted center-of-mass (CoM) calculations (mean ± SD; n = 34 hemi-bulbs from 17 sessions) were performed on the top 5% of clustered, active voxels for all odorants (AA—green; Nona—blue; 2HA—red; Lim—yellow) for both pulse sequences (solid line—CS-FLASH; dashed line—GE-EPI). (D) Euclidean distance was used to capture intra-mouse variability between CoM across scan days (mean ± SE; n = 7 mice) to characterize reproducibility of GL activation patterns.

Two-dimensional flat maps of GL activation were generated to better visualize spatial activation patterns of odorants. Flat maps further allow for more direct comparison with prior 2DG literature. Two scans were omitted due to the loss of the most caudal slice during group map normalization (Fig. 5; n = 17). Like GE-EPI data, all flat maps showed similar activation patterns between the left and right OB; therefore, the two hemispheres were averaged for the current analysis (data not shown). The CS-FLASH flat maps were complementary to our previous GE-EPI data (Supplementary Fig. 9) and other 2DG findings. Specifically, CS-FLASH captured the narrow activation of 2HA in the dorsolateral and dorsomedial regions (Fig. 5B) that roughly correspond with the M72 glomeruli, for which 2HA is a potent activator (Smear et al., 2013). Nona also activated the ventromedial and ventrolateral regions, while AA and Lim activated lateral regions and other ventromedial regions (Johnson et al., 1998, 2002, 2009). All GL flat maps produced two discrete activation regions in the dorsolateral and ventromedial bulb.

While qualitative assessment of the odor-evoked flat maps appeared to be spatially discrete, we quantitatively assessed their variability between mice (Fig. 5C). For this, we compared the weighted center of masses (CoMs) of the z-scores between odors. Individual ROIs were used to calculate the CoMs for each of the two discrete activation loci (Fig. 5B, dotted white lines). A one-way, repeated measures MANOVA with Šidák’s multiple comparisons post hoc tests was used to assess the spatial discreteness of the four odor-activation flat maps. While all four odors produced unique CoMs in both ROIs for GE-EPI (Fig. 5C and D, dotted lines), CS-FLASH (solid line) produced unique activation patterns in all ROIs except between AA and Lim in ROI2 (in-plane CoM AA-Lim p = 0.998 and out-of-plane CoM AA-Lim p = 0.560; Supplementary Tables 4 and 6).

To examine the reproducibility of the odor-evoked flat maps for each mouse (n = 7 mice) acquired on different scans days (2–3 scan days per mouse), we compared the Euclidean distance of the CoMs for each ROI across the different days. The average intra-mouse distance between CoMs for CS-FLASH was 275.9 ± 182.7 µm for ROI1 (dorsolateral) and 359.7 ± 193.9 µm for ROI2 (ventromedial). Nona had the smallest differences in CoM locations for ROI1 and ROI2 (164.3 ± 132.2 µm and 170.9 ± 128.6 µm, respectively), while AA had the greatest (357.7 ± 99.2 µm and 491.0 ± 152.1 µm). Compared with GE-EPI, CS-FLASH values had greater intra-mouse CoM variability in the dorsolateral area (ROI1), but not the ventromedial area (ROI2; paired Students t-test, p = 0.014 and p = 0.67, respectively). Interestingly, while GE-EPI had greater reproducibility for ROI1 than for ROI2, CS-FLASH has similar reproducibility values for both ROI1 and ROI2, as more of its mean values were closer to the identity line. The average intra-mouse in-plane versus out-of-plane differences for CS-FLASH were 173.2 ± 122.5 µm and 155.4 ± 189.2 µm for ROI1, and 255.4 ± 153.3 µm and 198.2 ± 173.5 µm for ROI2, respectively. Most odors for CS-FLASH had similar in-plane and out-of-plane differences in CoM (Supplementary Fig. 10), except for AA/Nona-ROI1 out-of-plane differences (Kruskal–Wallis test and a Wilcoxon Rank Sum post hoc test, p < 0.01) in which Nona had no out-of-plane variability. In-plane and out-of-plane differences in CoM for CS-FLASH were comparable with GE-EPI, except for ROI1 in-plane differences (Wilcoxon Rank Sum, p < 0.01) in which GE-EPI had less in-plane variability.

These results highlight CS-FLASH as a comparable pulse sequence with GE-EPI for acquiring spatially unique activation maps in awake rodents with a few key considerations: (1) qualitatively, CS-FLASH has better spatial focality (Fig. 3) and less smoothing of activation maps, potentially improving high-resolution imaging; (2) CS-FLASH had a lower dynamic range of t-values or response sensitivity; (3) within the OB, CS-FLASH tended to have a higher degree of intra-mouse variability but with less variability between the two ROIs.

### 3.4 Layer-dependent fMRI time series: CS-FLASH versus GE-EPI

**Fig. 6.**
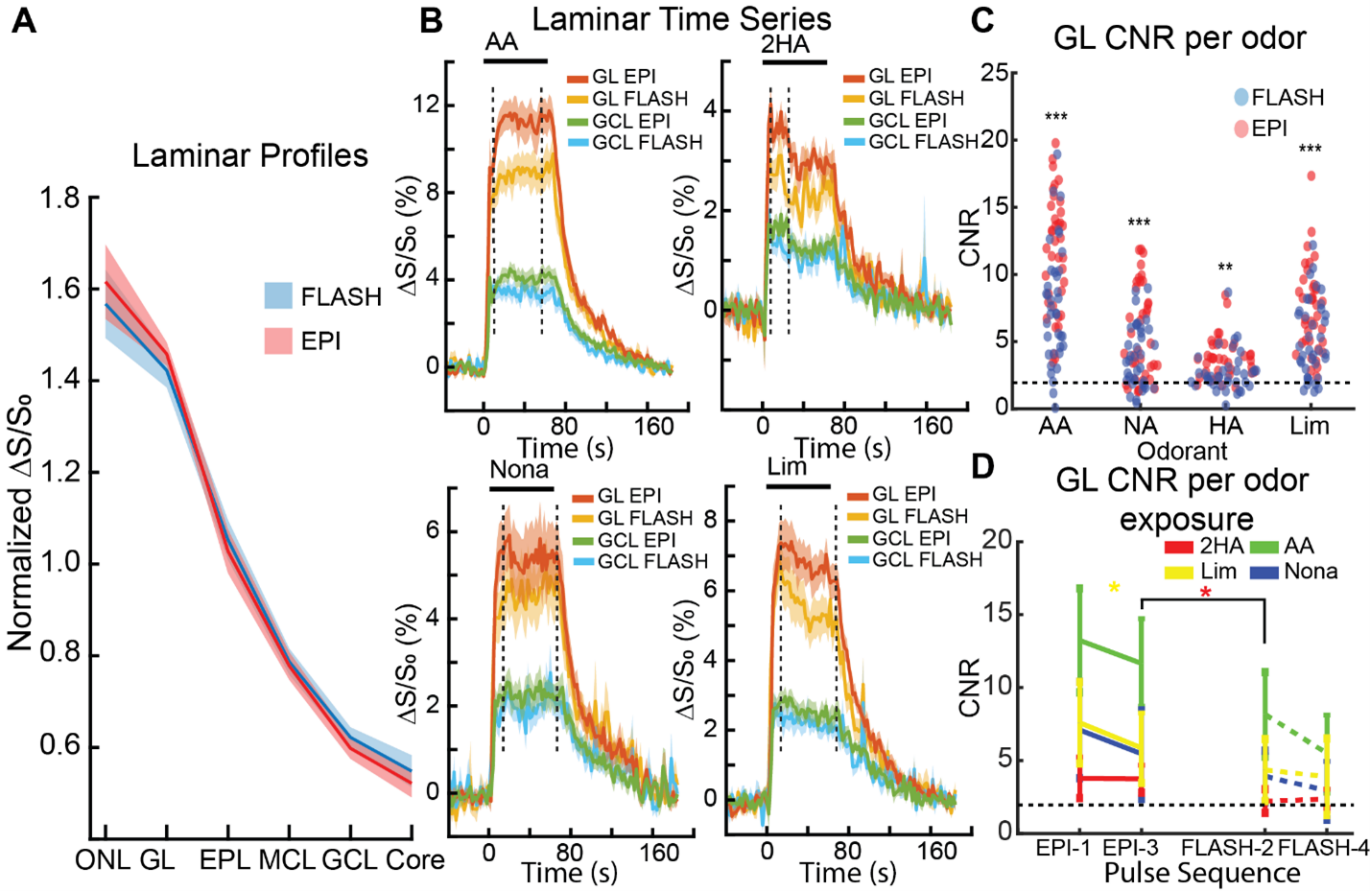
Laminar CBVw fMRI responses (A) Relative normalized fMRI response (ΔS/S_0_) to odor-evoked activation of the OB to show layer-specific CBVw responses (n = 19, no threshold applied). The value 1 represents the average laminar response of each odor. ONL—olfactory nerve layer, GL—glomerular layer, EPL—external plexiform layer, MCL—mitral cell layer, GCL—granule cell layer. (B) GE-EPI versus CS-FLASH time series between the superficial GL and deeper GCL. Time series were normalized to the baseline (full baseline not shown, -120–0 s and 160–184 s) for each session and averaged across sessions. Horizontal bars represent odor exposure (0–64 s). All graphs are mean ± SEM. (C) GL contrast-to-noise ratio (CNR) for GE-EPI (red dots) and CS-FLASH (blue dots) for each odor (n = 38 per odor & per pulse sequence). Dotted black line represents a t-statistic of 1.96 (i.e., p < 0.05). Wilcoxon Rank Sum: **p < 0.01, ***p < 0.001. (D) GL CNR comparison per pulse sequence. Note, pulse sequence order was GE-EPI (1) → CS-FLASH (2) → GE-EPI (3) → CS-FLASH (4). One-way repeated measures ANOVA with Šidák’s post hoc test for multiple comparisons: *p < 0.05. Asterisk color indicates the odor that was significant.

We previously showed that odor-evoked fMRI laminar profiles for both GE-EPI in awake mice and CS-FLASH in anesthetized rats were layer dependent, with the greatest signal changes in superficial layers that decreased with laminar depth (Poplawsky et al., 2015, 2023). Here, direct comparisons of these fMRI sequences are made. First, the mean fMRI signal change of all voxels in each layer was normalized by the mean signal change across all six layers for each odor to remove fMRI strength differences between odors and acquisition techniques. The laminar profiles of the four odors were not significantly different from each other using two-way repeated measures ANOVAs with Geisser–Greenhouse’s correction (Supplementary Fig. 11; GE-EPI: F(1.944, 34.98) = 3.49 x 10^-13^, p > 0.99; CS-FLASH: F(1.706, 30.70) = 6.66 x 10^-14^, p > 0.99), so the laminar profiles were averaged across odors for each fMRI sequence (Fig. 6A). These mean laminar profiles were not significantly different between GE-EPI and CS-FLASH using a two-way repeated measures ANOVA (F(1, 18) = 1.315 x 10^-13^, p > 0.99), suggesting that *relative* laminar responses were nearly the same between these two fMRI sequences.

To examine the *absolute* percentage signal change differences, we calculated the layer-dependent time series by averaging all voxels without thresholding in the sensory input (GL) and feedback (GCL) layers. Here, the odor-evoked responses were consistently greater for GE-EPI than for CS-FLASH (Fig. 6B). To examine this further, we calculated the GL CNR for each individual odor run. Here, individual GE-EPI trials tended to have higher CNR than CS-FLASH for all four odors (Fig. 6C). However, since fMRI signal attenuation occurs in awake mice with repeated odor exposure (see fig. 6 by Poplawsky et al., 2023) and GE-EPI often preceded CS-FLASH in our interleaved design (Fig. 1A), olfactory adaptation may account for this difference. Therefore, we compared the CNRs of the individual runs for fMRI sessions with the exact sequence order of GE-EPI → CS-FLASH → GE-EPI → CS-FLASH (n = 14). Using a one-way repeated measures ANOVA with Geisser–Greenhouse’s correction for each odor (AA: F(2.553, 33.19) = 10.00, p = 0.0002; Nona: F(2.227, 28.95) = 7.928, p = 0.0013; 2HA: F(2.039, 26.51) = 7.756, p = 0.0021; Lim: F(1.718, 22.33) = 7.551, p = 0.0044), this run-by-run analysis indicated that olfactory adaptation likely does not fully explain the greater CNR for GE-EPI since the 3^rd^ odor exposure (GE-EPI) was significantly greater (2HA: p = 0.0354; Šídák’s multiple comparisons tests) or trended greater (AA: p = 0.0640; Nona: p = 0.0858; Lim: p = 0.0627) than the 2^nd^ (CS-FLASH) (Fig. 6D). Instead, other factors may have more significant contributions, most notably the TR (1,000 ms for GE-EPI; 125 ms for CS-FLASH).

## 4 Discussion

In the current study, we compared odor-evoked CBVw fMRI responses obtained with CS-FLASH or GE-EPI in the awake mouse olfactory bulb to examine the impact of functional pulse sequence selection on fMRI motion and activation metrics (Poplawsky et al., 2023). We found that (1) CS-FLASH had comparable motion characteristics with prior literature, but had features unique to the pulse sequence, such as decreased median motion compared with GE-EPI with smaller motions but a susceptibility to image corruption during large motion events; (2) CS-FLASH can map unique odor activation patterns that are specific to the odor identities but was less sensitive than GE-EPI; and (3) CS-FLASH had comparable laminar profiles and time series as GE-EPI. Collectively, the current study shows that CS-FLASH is a viable alternative pulse sequence to GE-EPI that can reliably capture fMRI activity at high resolutions in areas that may suffer from geometric distortions and signal dropout without extensive motion contamination.

### 4.1 Awake rodent fMRI using CS-FLASH

We previously used CS-FLASH to obtain reliable high-resolution images of activity within the anesthetized rat OB, a region with high air–tissue interfaces (Poplawsky & Kim, 2014; Poplawsky et al., 2015, 2021; Zong et al., 2014). The acquisition of a single echo per TR (125 ms; 2s effective TR) allows for reliable imaging with less image distortion or signal dropout in the OB compared with GE-EPI. While anesthesia helps mitigate motion confounds, it influences neural processing, vascular reactivity, and neurovascular coupling. Thus, we wanted to investigate whether we can obtain similar high-resolution layer-specific results when imaging awake mice. Awake rodent fMRI has emerged as a reliable imaging technique to non-invasively study brain activity (Chen et al., 2020; Cover et al., 2021; Mandino et al., 2024), with acquired data having higher sensitivity to hemodynamic and polysynaptic activity (Desai et al., 2011; Liang et al., 2015).

#### 4.1.1 Motion characteristics of awake fMRI CS-FLASH

While implementation of novel MRI compatible restraint devices and acclimation protocols significantly reduces motion, scans are not devoid of it. How motion impacts the fidelity of the MRI data depends on the acquisition parameters of the pulse sequence. Compared with traditional pulse sequences, CS-FLASH relies on compressed sensing to accelerate fMRI volume acquisitions (Zong et al., 2014). Per effective TR (TR_E_; 2s), only ¼ of k-space (i.e., 16 lines; 1 line per 125 ms) is acquired, with the center 6 k-space lines always being acquired and the other 10 lines randomly sampled from the remaining lines of k-space per TR_E_. Volume reconstruction then relies on temporal redundancies to generate images of the OB. The acquisition of individual lines of k-space by CS-FLASH produces higher fidelity images than GE-EPI with less signal dropout and blurring caused by T2* decay. These sharper images may explain why CS-FLASH has smaller total FD values (Fig. 2B) for relatively small motions. However, CS-FLASH acquired the k-space lines evenly throughout the effective TR (2s), unlike GE-EPI that quickly acquired its lines within ~400 ms or 20% of the effective TR. This increases the probability that CS-FLASH will capture larger motions that fully corrupt the image volume (Fig. 2B inset and Supplementary Fig. 2). Consistent with our prior study, motion appeared to occur sporadically throughout the fMRI runs, except for at the beginning (~1 TR; 2s) and immediately following odor onset and cessation (~1–2 TR; 2–4s). Similarly, this preferentially impacts the initial period of the hemodynamic response, weighing the GLM toward the longer plateau period. This biases the data toward capturing local micro-vessel hemodynamic activity that is more specific to neuronal activity (Jin & Kim, 2008).

CS-FLASH, compared with GE-EPI, is subjectively a quieter pulse sequence, which we initially assumed would result in lower amounts of motion. Although CS-FLASH had smaller total FD values (Fig. 2B), body motion measured by a pressure sensor showed comparable motion between CS-FLASH and GE-EPI. These results corroborate prior work reporting no physiological or behavioral stress differences between awake head-fixed rodents exposed to MRI pulse sequences with sound levels up to 120 dB and unexposed control mice (Almeida et al., 2022).

While CS-FLASH motion parameter estimates were comparable with prior studies (Chen et al., 2020; Cover et al., 2021; Han et al., 2019; Poplawsky et al., 2023), standard FD methods to characterize high-motion volumes may censor out usable data. FD is calculated as the Euclidean distance between successive fMRI volumes as a first derivative. However, we determined that 88% of censored motion events were sporadic and quickly returned to baseline motion levels. Therefore, with FD censoring, volumes with high spatial correlation to the mean baseline image (i.e., usable volumes, r_spatial_ > 0.95) are often censored when they follow a high motion volume with poor spatial correlation (r_spatial_ < 0.95). In the current study, we utilized FD as our primary method for detecting high motion volumes for direct comparison with our prior publication. However, a mixed spatial correlation and FD approach may be more appropriate to recover usable censored volumes, thus increasing the degrees of freedom. While this approach did not improve the statistical strength of our current activation maps (Supplementary Fig. 8), this is likely due to our long odor stimulation periods in which over-censoring does not significantly impact the results. However, for shorter stimuli, doubling the number of censored frames could substantially impact detection sensitivity. CS-FLASH, more so than GE-EPI, may require the use of alternative censoring techniques, such as spatial correlation-based censoring, to preserve statistical power.

A potential limitation of the current study is that the odor presentation and pulse sequence order were fixed with GE-EPI consistently occurring earlier in the scan session than CS-FLASH. This may have biased the CS-FLASH data toward more high-motion events if mice increased their motion with time in the scanner. However, we found no difference in motion characteristics between the first train of CS-FLASH scans and the last.

#### 4.1.2 Odor-evoked activation and flat maps

Odor-evoked activation maps for CS-FLASH had comparable spatial activation patterns as our prior GE-EPI findings (Poplawsky et al., 2023), which were also comparable with maps obtained with histological 2DG experiments (Johnson et al., 1998, 2002, 2009; Smear et al., 2013). Similar to GE-EPI, CS-FLASH is capable of producing high-resolution odor-specific activation patterns in the awake state. In-plane resolution of this study was 100 × 100 µm^2^ which approaches the size of a glomerulus. Therefore, it is important when resolving activity at the submillimeter scale that the data are devoid of motion artifacts that would reduce experimental sensitivity and specificity.

One approach we took to validate the uniqueness of our activation patterns was to characterize the percent of overlap of all four odors at increasing thresholds (i.e., top 10%, 5%, 2.5% and 1%; Suppl. Fig. 7). Consistent with the literature, 2HA had the highest spatial uniqueness with very limited overlap with the three other odors. 2HA is a potent activator of the M72 odorant receptor. Our spatial activation patterns were consistent with the histological labeling of the M72 odorant receptors in glomeruli on the dorsolateral and dorsomedial regions of the OB (Smear et al., 2013). Comparatively, AA and Lim had the lowest spatial uniqueness and largest spatial overlap among the odors used in this study. We next utilized CoM calculations to evaluate spatial uniqueness of activation patterns more quantitatively. Like 2DG studies, fMRI flat maps were generated from GL (Fig. 5). Consistent with our top threshold spatial overlap findings, 2HA and Nona had the most consistently unique CoMs for both the dorsolateral and ventromedial ROIs. Alternatively, AA and Lim had statistically unique activation patterns for the dorsolateral, but not ventromedial ROIs. Further, AA and Lim had greater degrees of spatial overlap in GE-EPI data, independent of thresholding, compared with CS-FLASH (ex. 8.1% vs. 4.9% AA ∩ Lim for GE-EPI and CS-FLASH, respectively).

Within mouse reproducibility of activation maps is also an important metric when evaluating robustness of an imaging technique. The underlying assumption in this instance is a mouse should produce a similar activation map across scans, which has been shown to occur in optogenetic fMRI experiments in awake rats (Liang et al., 2015). The Euclidean distance between CoM locations captures this variability and gives insight into the reliability of measuring spatially discrete activations. Overall, CS-FLASH had greater variability in odorants that more strongly activate the OB, with AA and Lim lying in the top right corner of Figure 4D, while Nona had the lowest variability. Further, the intra-mouse CoM location variability is 100–500 µm for ROIs 1 & 2, indicating that imaging with CS-FLASH generates reproducible activation maps of odorants. Alternatively, GE-EPI has improved reliability in ROI1 with an intra-mouse CoM range of 50–350 µm, but comparable CoM variability in ROI2 with a range of 100–600 µm. This variability in the awake state is expected to occur, especially over different scan days (Bergmann et al., 2020; Gutierrez-Barragan et al., 2022), and may be explained, in part, to inexact slice positioning or group normalization.

#### 4.1.3 CS-FLASH layer-dependent time-series analysis

An aim of our prior study was to examine whether layer-specific activity can be reliably imaged using CBVw fMRI in awake mice. We previously determined that GE-EPI could capture localized hemodynamic responses, making it a good pulse sequence to examine layer-specific neuronal activity in awake mice. Similar to our previous study, CS-FLASH also effectively measured layer-specific hemodynamic responses with the greatest responses in superficial input layers that decreased with laminar depth. Qualitatively, the average hemodynamic responses within the GL and GCL were very comparable in shape with differences predominately seen in the peak amplitudes (Fig. 6B). Quantitatively, individual odor exposures were often significantly detectable (Fig. 6C, CNR > 1.96), although 2HA had the most scans with a CNR < 1.96. While this may suggest that 2HA is less detectable, it may instead be due to calculating CNR from the whole GL. Of the four odors, 2HA had the most discrete, localized GL activation, especially when measured by CS-FLASH even when compared with GE-EPI; thus, there are substantial regions of inactive GL used in the CNR calculation.

### 4.2 Functional pulse sequences, CS-FLASH versus GE-EPI: advantages and considerations

Pulse sequence selection produces new considerations when designing and analyzing results from an awake fMRI experiment. For example, the nature of data acquisition can alter data processing pipelines to favor alternative strategies, such as the use of spatial correlation versus FD to preserve statistical power after censoring. An advantage of the current study is the use of a long odorant stimulus duration (32 TRs; i.e., 64 s) and use of contrast-enhanced CBVw fMRI for increased detection sensitivity and spatial specificity (Poplawsky et al., 2019a). However, the unique sensitivities of GE-EPI and CS-FLASH can be seen in the distribution of z-scores (Fig. 4C) and the average GL CNR (Fig. 6D), in which GE-EPI regularly outperformed CS-FLASH. In addition, GE-EPI is a standard sequence included on most MRI scanners, while CS-FLASH is not and may be technically challenging to implement. Therefore, GE-EPI may have more benefits as the choice to image at high spatial resolutions. However, the quality of our GE-EPI images (Fig. 1B) can be attributed to the short 7.5 ms TE used with contrast enhancement, localized bulb shimming, and a high-performance integrated shim system (Bruker BGA-12S2 HD, 10 A/channel, up to 660 mT/m). GE-EPI studies with endogenous BOLD contrast and longer TE’s, whole-brain imaging with global shimming, or lower performing shim systems may not produce images with sufficient quality, especially in mice. In such scenarios, CS-FLASH is still a viable alternative for such applications. Lastly, we used a ~1-min odor exposure and waited ~20 min between presentations of the same odor to provide ample functional contrast (Poplawsky et al., 2015; Poplawsky et al., 2019b), limit adaptation (Schafer et al., 2005; Zhao et al., 2016), and preserve the spatial pattern of activation to repeated odor stimulations (Schafer et al., 2006). However, it would be interesting to compare fMRI responses with much shorter odor stimuli (<10 s) and interstimulus intervals (<30 s) that are typical to rodent behavioral studies of olfaction.

## 5 Conclusions

In summary, the current study compared the traditional GE-EPI functional pulse sequence with an alternative CS-FLASH sequence to evaluate its reliability in high-resolution CBVw fMRI of the awake rodent olfactory bulb. GE-EPI and CS-FLASH both produced similar odor-specific spatial activation maps with minimal motion contamination and geometric distortions. However, GE-EPI consistently outperformed CS-FLASH in terms of detection sensitivity. Furthermore, GE-EPI is a commercial pulse sequence on many pre-clinical MRI scanners, requiring no advanced technical knowledge beyond what is standard in the field, making it easier to readily implement. However, CS-FLASH did produce sharper images and is a sufficient alternative when quality GE-EPI images are not feasible.

## Supplementary Material

Supplementary Material

## Data Availability

Data and scripts for analysis will be made available from the corresponding author on reasonable request due to the need for a formal data sharing agreement. The 3D files of the mouse cradle used in the current study can be found at https://github.com/neuroimlabpitt/Olfactory-fMRI.
