# Supplementary material for: Awake rodent fMRI: Gradient-echo echo planar imaging versus compressed-sensing fast low-angle shot
Source: Imaging Neurosci (Camb). 2025 Jan 2;3:imag_a_00406. doi: 10.1162/imag_a_00406 (PMC12330865; doi:10.1162/imag_a_00406)
Supplement: Supplementary Material [file imag_a_00406-supp.pdf]

Awake rodent fMRI: gradient-echo echo planar imaging versus compressed-sensing fast low angle shot

Supplementary figures and tables

Christopher Cover[1,2], Sujatha Reddy[1], Alberto Vazquez[1,2], Mitsuhiro Fukuda[1], Alexander J. Poplawsky[1*]

Institutions:

1. Department of Radiology, University of Pittsburgh, McGowan Institute for Regenerative Medicine Building, 3025 E. Carson St., rm. 159, Pittsburgh, PA, 15203, United States
2. Department of Bioengineering, University of Pittsburgh, Pittsburgh, PA, United States

[*] **Corresponding author:**

E-mail address: ajp94@pitt.edu (A. J. Poplawsky)

Supplemental Figures

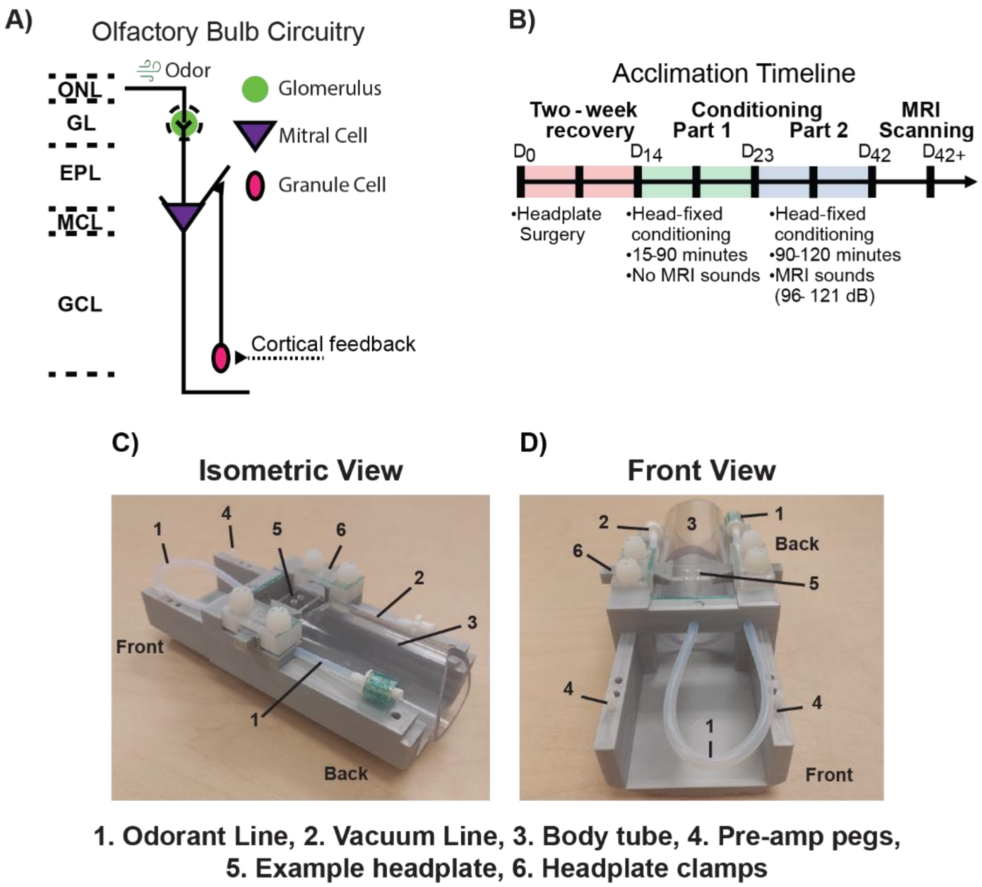

Supplemental Figure 1. A) Schematic overview of the laminar olfactory bulb circuit, highlighting the prominent cell types. ONL – olfactory nerve layer, GL – glomerular layer, EPI – external plexiform layer, MCL – mitral cell layer, and GCL – granule cell layer. B) Surgical and acclimation protocol to condition awake mice to head fixation in the MRI scanner. C) Isometric view and D) front view of the MRI compatible 3D-printed head restraint system with body restraint tube and Teflon odorant tube.

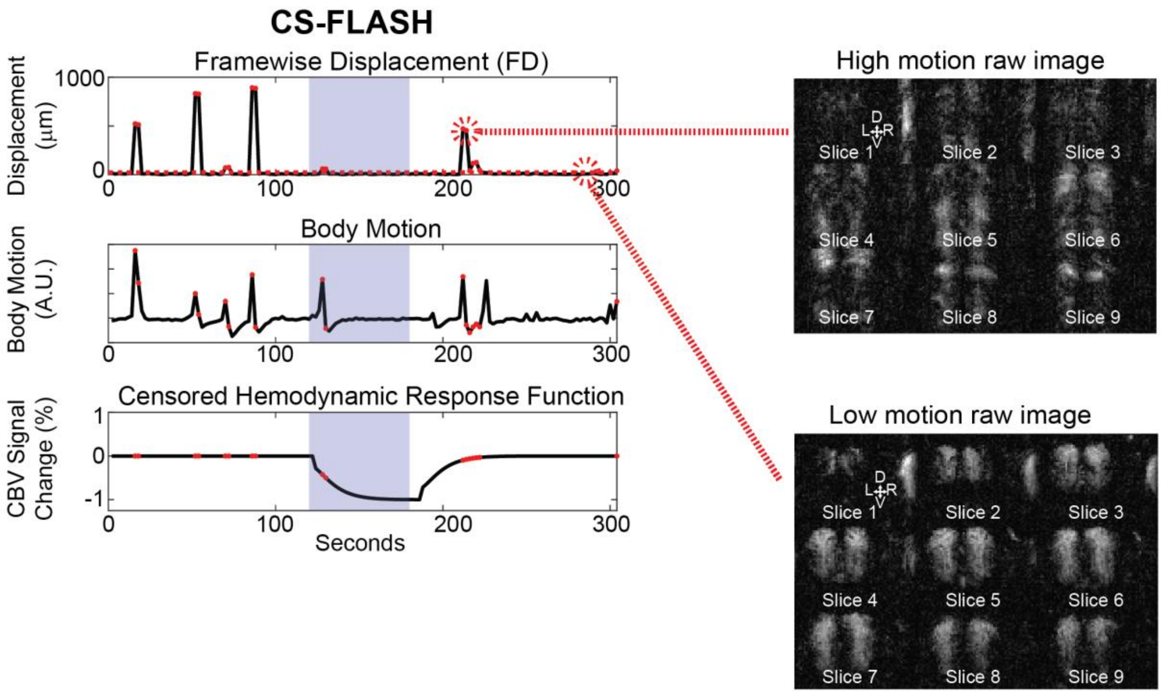

Supplemental Figure 2. Example traces of CS-FLASH framewise displacement and censoring of high motion data points (>25 μm, red dots) with associated body motion measured with a pneumatic respiratory pillow sensor during an fMRI scan. Additionally, an example cerebral blood volume-weighted (CBVw) hemodynamic response function with censoring. Traces are paired with example raw CS-FLASH images during high (top) and low (bottom) motion frames. Note the high level of image corruption during high motion.

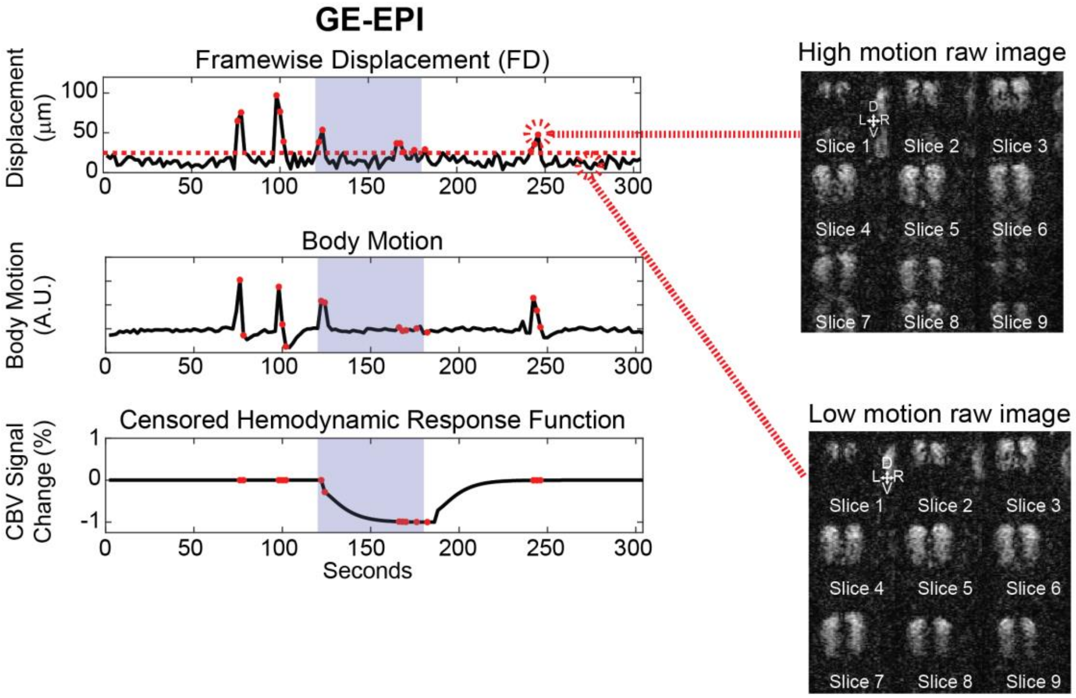

Supplemental Figure 3. Example traces of GE-EPI framewise displacement and censoring of high motion data points (>25 µm, red dots) with associated body motion measured with a pneumatic respiratory pillow sensor during an fMRI scan. Additionally, an example cerebral blood volume-weighted (CBVw) hemodynamic response function with censoring. Traces are paired with example raw GE-EPI images during high (top) and low (bottom) motion frames. Note the aliasing associated with high motion.

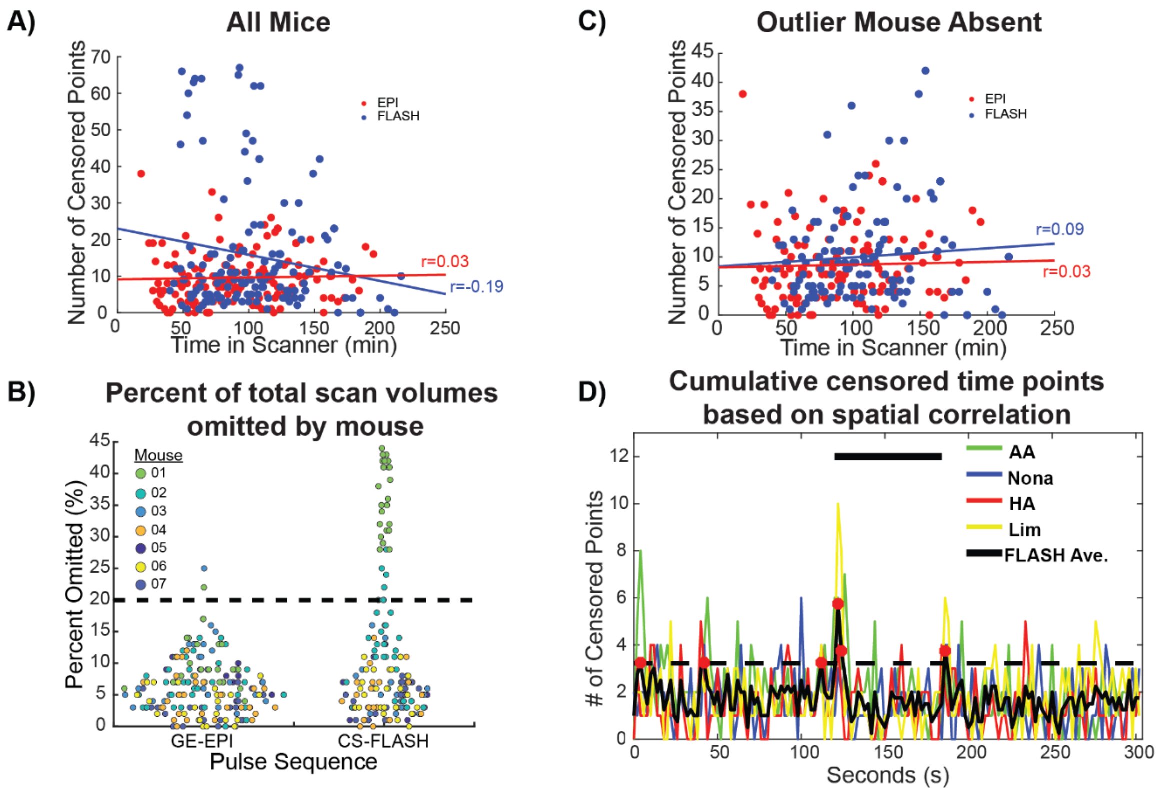

Supplemental Figure 4. A) Linear fit of GE-EPI (red) and CS-FLASH (blue) number of censored volumes over length of scan session. Time at 0 min represents the time stamp associated with the initial localizer sequence when the mice first entered the magnet. B) Percent of total scan volumes omitted grouped by mouse. Most of the highly censored scanned volumes originated from the same mouse (01), there was no association with odor type. C) Same plot as seen in (A) but with the removal of the one outlier mouse (01) that was highly censored. D) Cumulative censored time points for CS-FLASH over the length of the scan based on spatial correlation ($r < 0.95$ was censored) for all odors. Similar to censoring based on framewise displacement (>25 µm), there was increased censoring at the beginning of the scan and at odorant state changes.

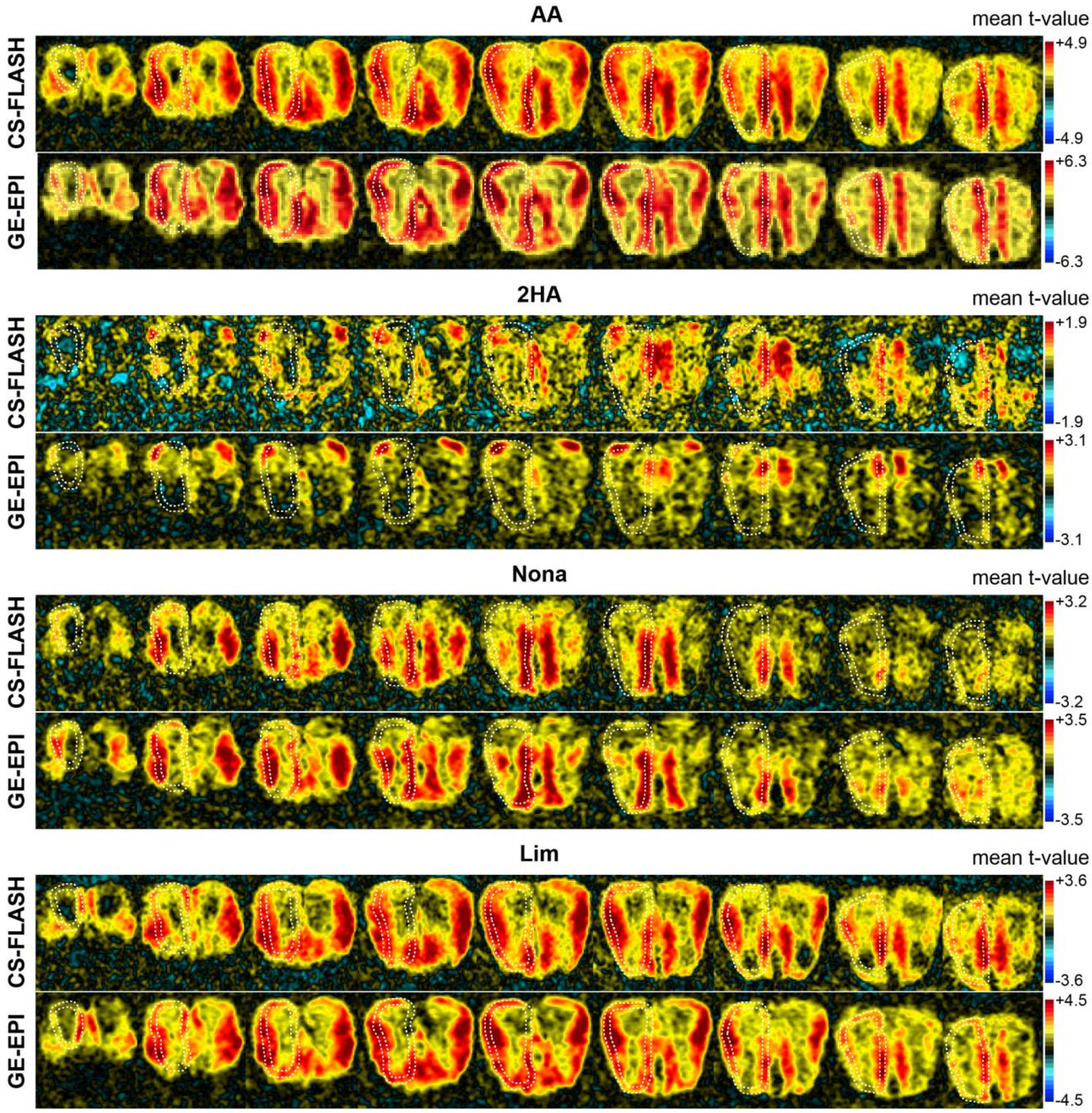

Supplemental Figure 5. Mean t-value group activation maps for CS-FLASH and GE-EPI. T-maps generated from *3dREMLfit* were normalized and averaged (n = 19 sessions, 7 mice, 2-3 sessions/mouse) for each odorant and are displayed without a threshold applied. Rostral OB slices are on the left and progress caudally to the right. The input layer, GL, is outlined with dotted white lines. Mean t-maps are for contrast purposes only and are not intended for statistical inference.

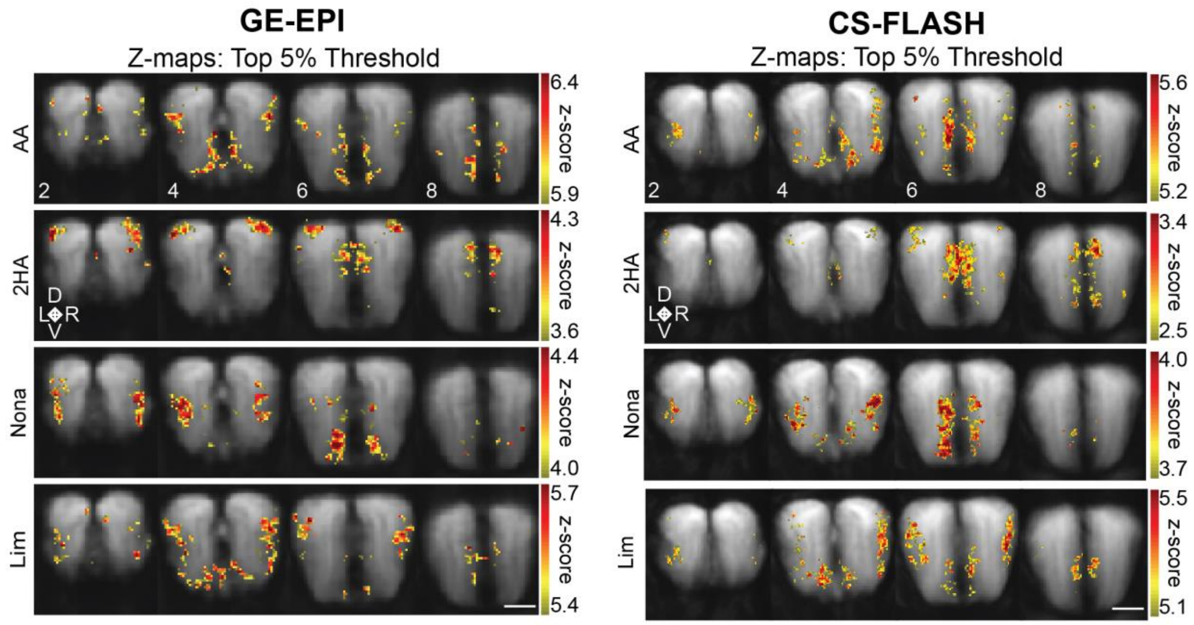

Supplemental Figure 6. Odor specific group activation maps, GE-EPI vs. CS-FLASH. Group Z-maps were calculated using *3dMEMA* with FDR correction (n = 19 sessions, 7 mice, 2-3 sessions/mouse), activation map for the top 5% of active voxels. Dynamic warm color-bar ranges were used to match the color distributions of z-scores across odors and sequences. More rostral OB slices are indicated with a lower slice number (see AA panel) and progress caudal. Clusters had a minimum cluster size of 10 voxels. GE-EPI q-value thresholds – AA: $q < 5.5 \times 10^{-9}$; 2HA: $q < 4.9 \times 10^{-4}$; Nona: $q < 7.6 \times 10^{-5}$; Lim: $q < 8.9 \times 10^{-8}$. CS-FLASH q-value thresholds – AA: $q < 2.70 \times 10^{-7}$; 2HA: $q < 0.018$; Nona: $q < 3.16 \times 10^{-4}$; Lim: $q < 5.57 \times 10^{-7}$. 1-mm scale bars. D – dorsal, V – ventral, L – left, R – right.

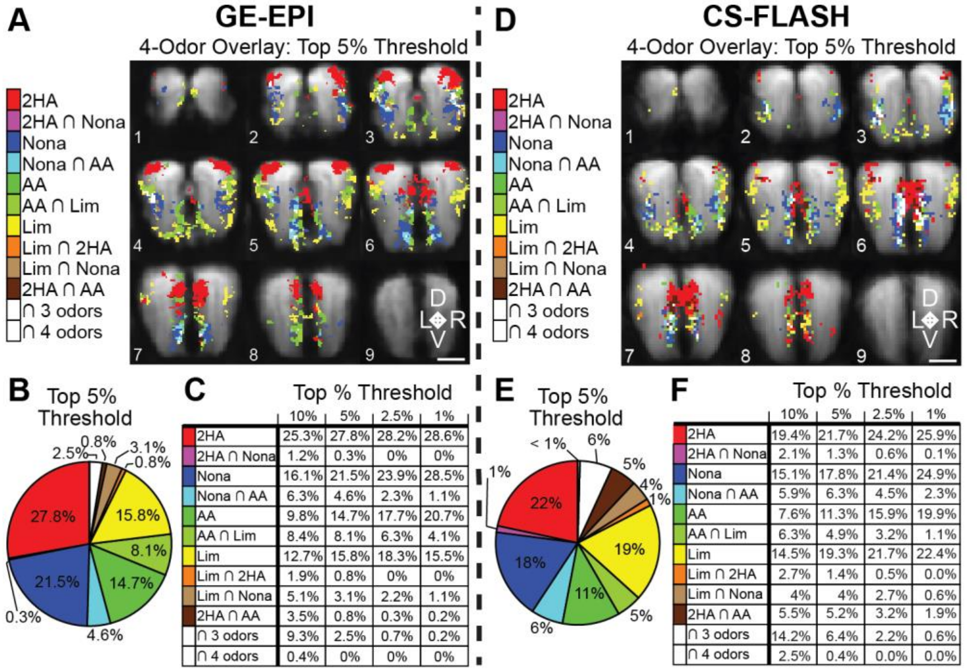

C

	Top % Threshold 10%	5%	2.5%	1%
2HA	25.3%	27.8%	28.2%	28.6%
2HA ∩ Nona	1.2%	0.3%	0%	0%
Nona	16.1%	21.5%	23.9%	28.5%
Nona ∩ AA	6.3%	4.6%	2.3%	1.1%
AA	9.8%	14.7%	17.7%	20.7%
AA ∩ Lim	8.4%	8.1%	6.3%	4.1%
Lim	12.7%	15.8%	18.3%	15.5%
Lim ∩ 2HA	1.9%	0.8%	0%	0%
Lim ∩ Nona	5.1%	3.1%	2.2%	1.1%
2HA ∩ AA	3.5%	0.8%	0.3%	0.2%
∩ 3 odors	9.3%	2.5%	0.7%	0.2%
∩ 4 odors	0.4%	0%	0%	0%

F

	Top % Threshold 10%	5%	2.5%	1%
2HA	19.4%	21.7%	24.2%	25.9%
2HA ∩ Nona	2.1%	1.3%	0.6%	0.1%
Nona	15.1%	17.8%	21.4%	24.9%
Nona ∩ AA	5.9%	6.3%	4.5%	2.3%
AA	7.6%	11.3%	15.9%	19.9%
AA ∩ Lim	6.3%	4.9%	3.2%	1.1%
Lim	14.5%	19.3%	21.7%	22.4%
Lim ∩ 2HA	2.7%	1.4%	0.5%	0.0%
Lim ∩ Nona	4%	4%	2.7%	0.6%
2HA ∩ AA	5.5%	5.2%	3.2%	1.9%
∩ 3 odors	14.2%	6.4%	2.2%	0.6%
∩ 4 odors	2.5%	0.4%	0.0%	0.0%

Supplemental Figure 7. Odor specific group maps spatial overlap, GE-EPI vs. CS-FLASH. A) GE-EPI and D) CS-FLASH, 4-odor overlay map where activations for each odor (top 5% threshold) and their intersections (∩) with other odors are color-coded, respectively. B) GE-EPI, and E) CS-FLASH, proportion of each odor combination relative to all activations for the top 5% of active voxels. C) GE-EPI and F) CS-FLASH, proportions for other top 10%, 5%, 2.5%, and 1% thresholds. 1-mm scale bars. D – dorsal, V – ventral, L – left, R – right.

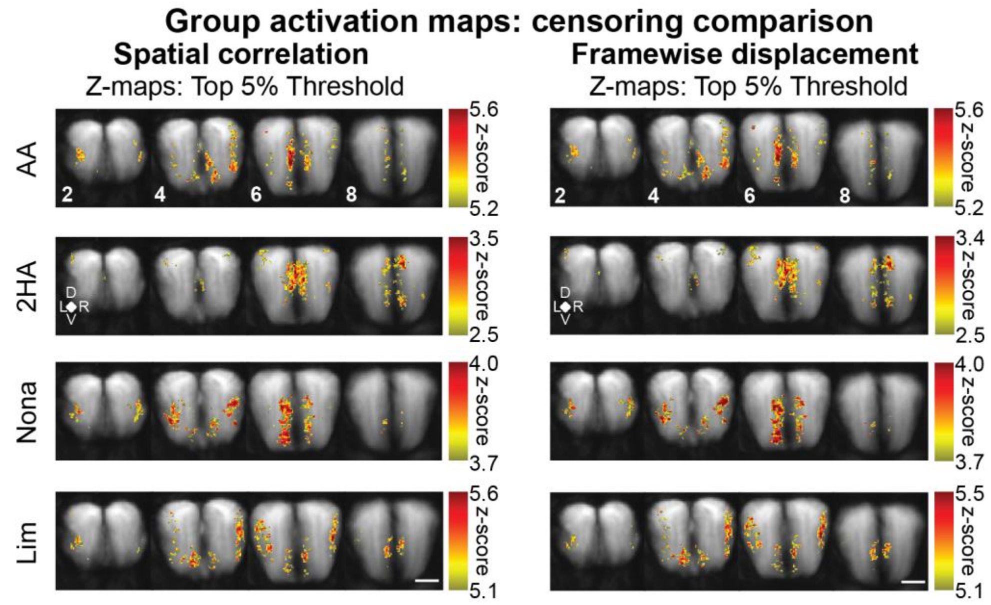

Supplemental Figure 8. CS-FLASH group odorant activation maps with spatial correlation censoring only. Individual scans were censored using spatial correlation (left) or framewise displacement (right) of the mean scan volume with each individual volume. Volumes with r < 0.95 were censored and removed from analysis. Group CS-FLASH z-maps were calculated using *3dMEMA* with FDR correction (n = 19 sessions, 7 mice, 2-3 sessions/mouse), activation map for the top 5% of active voxels (AA_{spat}: $q < 2.71 \times 10^{-7}$, AA_{FD}: $q < 2.70 \times 10^{-7}$; $2HA_{spat}$: $q < 0.019$, $2HA_{FD}$: $q < 0.018$; $Nona_{spat}$: $q < 2.9 \times 10^{-4}$, $Nona_{FD}$: $q < 3.16 \times 10^{-4}$; Lim_{spat}: $q < 4.81 \times 10^{-7}$, Lim_{FD}: $q < 5.57 \times 10^{-7}$) with a minimum cluster of 10 voxels. Dynamic warm color-bar ranges were used to match the color distributions of z-scores across odors and analysis methods. More rostral OB slices are indicated with a lower slice number (see AA panel) and progress caudally. 1-mm scale bars. D – dorsal, V – ventral, L – left, R – right.

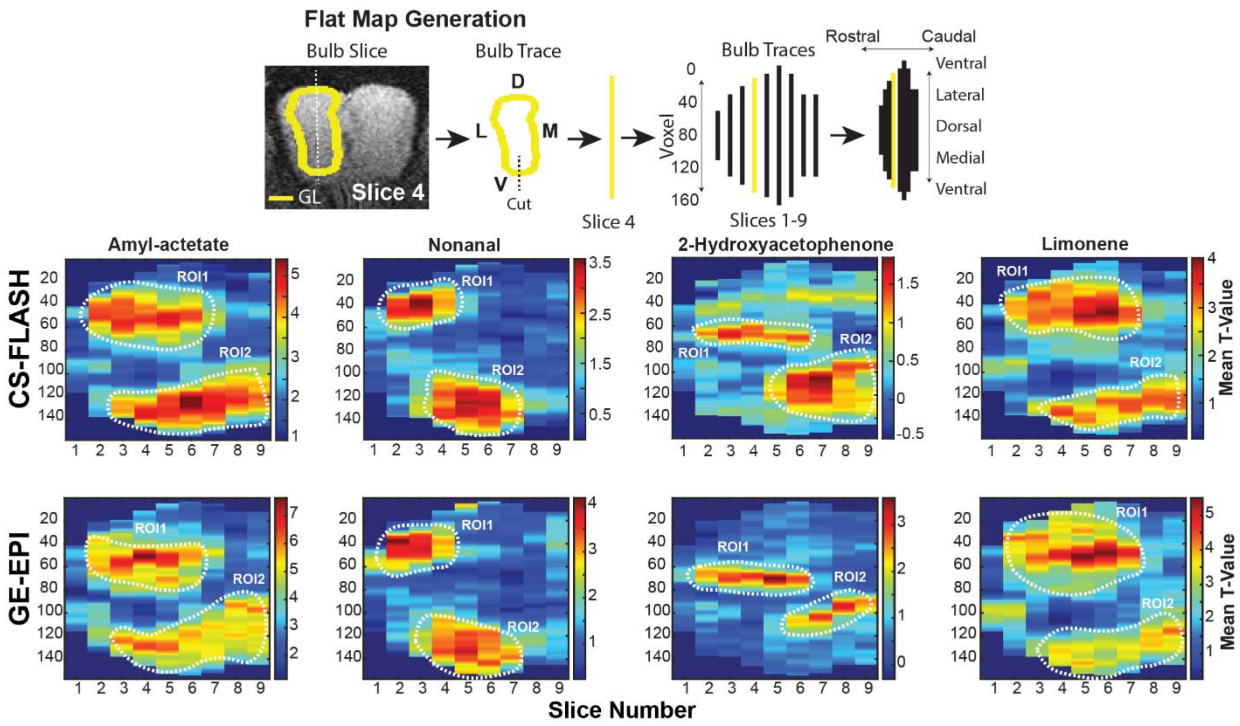

Supplemental Figure 9. Olfactory bulb odorant flat map comparison CS-FLASH vs. GE-EPI. (Top) Example overview of flat map generation from the glomerular layer (yellow) of slice 4. (Middle – CS-FLASH) and (Bottom – GE-EPI) Individual session t-maps were flattened (n = 17) and maps from both hemispheres were averaged (n = 34). Regions-of-interest (ROIs) 1 and 2 were outlined (dotted white line). Vertical scale is in-plane voxel number (50 µm/voxel); horizontal scale is slice number (300 µm/slice).

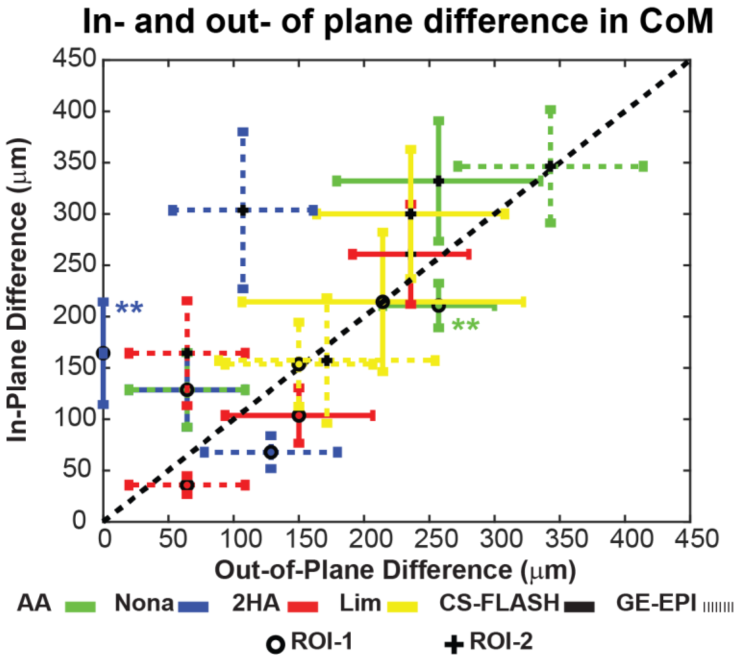

Supplemental Figure 10. In-plane versus out-of-plane contributions to intra-mouse Euclidean distance variability for CS-FLASH (solid line) and GE-EPI (dashed line). Dotted black line represents the identity line. Graph is mean ± SEM. Kruskal-Wallis test with Šidák's post-hoc test for multiple comparisons, and a Wilcoxon Rank sum with corrected p-value: ** $p < 0.01$. Assigned significance between Nona & AA in ROI1 out-of-plane difference.

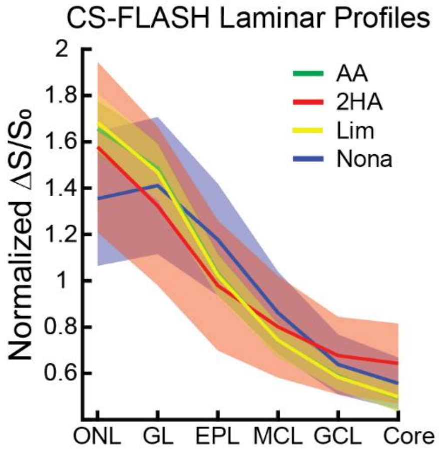

Supplemental Figure 11. Normalized layer-specific CBVw fMRI responses ($\Delta S/S_0$) were calculated across layers to show the relative laminar distribution of odor-evoked activations for each odor (n = 19, no threshold applied). The value of 1.0 represents the average laminar response for each odor.

Supplemental Tables

Supplementary Table 1. CS-FLASH motion parameter estimates between odors

Kruskal-Wallace Test, Bonferroni correct $p<0.0125$

**Motion parameter**	**Chi-squared (*df*; 161)**	**Sig.(*p*)**
Roll	4.11	0.249
Pitch	1.9	0.594
Yaw	1.23	0.746
X	0.9	0.827
Y	4	0.262
Z	3.45	0.327

Supplementary Table 2. CS-FLASH vs. GE-EPI motion parameter estimates

Kruskal-Wallace Test, Bonferroni correct $p<0.0125$

**Motion parameter**	**Chi-squared (*df*; 322)**	**Sig. (*p*)**
Roll	20.06	0.006**
Pitch	2.56	0.915
Yaw	11.37	0.123
X	1.33	0.988
Y	11.19	0.131
Z	92	0.239

Supplementary Table 3. Test of Between-Subject Effects at Flat Map Center-of-Mass ROI2

**Source**	**Dependent Variable**	**Type III Sum of Squares**	***df***	**Mean Square**	***F***	**Sig. (*p*)**	**Partial Eta Squared**
Corrected Model	In-plane	5994.515	3	1998.172	160.515	<0.001	0.883
	Out-of-plane	11.338	3	3.779	12.166	<0.001	0.364
Intercept	In-plane	192179.779	1	192179.779	15437.950	<0.001	0.996

	Out-of-plane	911.779	1	911.779	2934.959	<0.001	0.979
Fix-factor Odors	In-plane	5994.515	3	1998.172	160.515	<0.001	0.883
	Out-of-plane	11.338	3	3.779	12.166	<0.001	0.363
Error	In-plane	796.706	64	12.449			
	Out-of-plane	19.882	64	0.311			
Total	In-plane	198971.000	68				
	Out-of-plane	943.000	68				
Corrected Total	In-plane	6791.221	67				
	Out-of-plane	31.221	67				

[a.] R squared = 0.883 (Adjusted R Squared = 0.877),[b.] R squared = 0.363 (Adjusted R Squared = 0.333)

Supplementary Table 4. Univariate Effects at Flat Map Center-of-Mass ROI1, Multiple Comparisons Analysis, CS-FLASH

**Dependent Variables**	**Fix Factor 1 (I)**	**Fix Factor 2 (J)**	**Mean Difference (I-J)**	**Std. Error**	**Sig. (*p*)**	**95% Confidence Interval Lower Bound**	**95% Confidence Interval Upper Bound**
In-plane center-of-motion location	AA	Nona	9.4118*	1.23748	<0.001	6.1263	12.6973
		HA	-16.6471*	1.23748	0.000	-19.9325	-13.3616
		Lim	5.8235*	1.23748	<0.001	2.4639	9.1831
	Nona	AA	-9.4118*	1.23748	<0.001	-12.6973	-6.1263
		HA	-26.0588*	1.23748	0.000	-29.3443	-22.7733
		Lim	-3.5882*	1.23748	0.030	-6.9478	-0.2286
	HA	AA	16.6471*	1.23748	0.000	13.3616	19.9325
		Nona	26.0588*	1.23748	0.000	22.7733	29.3443

		Lim	22.4706*	1.23748	0.000	19.1110	25.8302
	Lim	AA	-5.8235*	1.23748	<0.001	-9.1831	-2.4639
		Nona	3.5882*	1.23748	0.030	0.2286	6.9478
		HA	-22.4706*	1.23748	0.000	-25.8302	-19.1110
Out-of-plane center-of-motion location	AA	Nona	0.7647*	0.20271	0.001	0.2457	1.2837
		HA	-0.3529	0.20271	0.351	-0.8720	0.1661
		Lim	-0.9412*	0.20271	<0.001	-1.4915	-0.3909
	Nona	AA	-0.7647*	0.20271	0.001	-1.2837	-0.2457
		HA	-1.1176*	0.20271	<0.001	-1.6367	-0.5986
		Lim	-1.7059*	0.20271	<0.001	-2.2562	-1.1556
	HA	AA	0.3529	0.20271	0.351	-0.1661	0.8720
		Nona	1.1176*	0.20271	<0.001	0.5986	1.6367
		Lim	-0.5882*	0.20271	0.030	-1.1386	-0.0379
	Lim	AA	0.9412*	0.20271	<0.001	0.3909	1.4915
		Nona	1.7059*	0.20271	<0.001	1.1556	2.2562
		Lim	0.5882	0.20271	0.030	0.0379	1.1386

AA – Amyl-acetate, Nona – nonanal, HA – 2-hydroxactephenone, Lim – Limonene

The error term is Mean Square (Error) = 0.311

*The mean difference is significant at the 0.05 level.

Supplementary Table 5. Test of Between-Subject Effects at Flat Map Center-of-Mass ROI2

**Source**	**Dependent Variable**	**Type III Sum of Squares**	***df***	**Mean Square**	***F***	**Sig. (*p*)**	**Partial Eta Squared**
Corrected Model	In-plane	2713.809[a]	3	904.603	30.560	<0.001	0.589
	Out-of-plane	32.044[b]	3	10.681	20.317	<0.001	0.488
Intercept	In-plane	1018710.721	1	1018710.721	34414.620	<0.001	0.988
	Out-of-plane	2681.309	1	2681.309	5100.112	<0.001	0.988
	In-plane	2713.809	3	904.603	30.560	<0.001	0.589

Fix-factor Odors	Out-of-plane	32.044	3	10.681	20.317	<0.001	0.488
Error	In-plane	1894.471	64	29.601			
	Out-of-plane	33.647	64	0.526			
Total	In-plane	1023319.00	68				
	Out-of-plane	2747.00	68				
Corrected Total	In-plane	4608.279	67				
	Out-of-plane	65.691	67				

[a.] R squared = 0.589 (Adjusted R Squared = 0.570),[b.] R squared = 0.488 (Adjusted R Squared = 0.464)

Supplementary Table 6. Univariate Effects at Flat Map Center-of-Mass ROI2, Multiple Comparisons Analysis, CS-FLASH

**Dependent Variables**	**Fix Factor 1 (I)**	**Fix Factor 2 (J)**	**Mean Difference (I-J)**	**Std. Error**	**Sig. (*p*)**	**95% Confidence Interval Lower Bound**	**95% Confidence Interval Upper Bound**
In-plane center-of-motion location	AA	Nona	-0.8235	1.82685	0.998	-5.8899	4.2428
		HA	14.2941*	1.82685	<0.001	9.2278	19.3605
		Lim	-2.8235	1.82685	0.558	-7.7832	2.1361
	Nona	AA	0.8235	1.82685	0.998	-4.2428	5.8899
		HA	15.1176*	1.82685	<0.001	10.0513	20.1840
		Lim	-2.000	1.82685	0.858	-6.9597	2.9597
	HA	AA	-14.2941*	1.82685	<0.001	-19.3605	-9.2278
		Nona	-15.1176*	1.82685	<0.001	-20.1840	-10.0513
		Lim	-17.1176*	1.82685	<0.001	-22.0773	-12.1580
	Lim	AA	2.8235	1.82685	0.558	-2.1361	7.7832

		Nona	2.0000	1.82685	0.858	-2.9597	6.9597
		HA	17.1176*	1.82685	<0.001	12.1580	22.0773
Out-of-plane center-of-motion location	AA	Nona	1.2941*	0.22877	<0.001	0.6730	1.9152
		HA	-0.9412*	0.22877	0.002	-1.6164	-0.2660
		Lim	-0.3529	0.22877	0.560	-0.9740	0.2681
	Nona	AA	-1.2941*	0.22877	<0.001	-1.9152	-0.6730
		HA	-2.2353*	0.22877	<0.001	-2.8564	-1.6142
		Lim	-1.6471*	0.22877	<0.001	-2.2681	-1.0260
	HA	AA	0.9412*	0.22877	0.002	0.2660	1.6164
		Nona	2.2353*	0.22877	<0.001	1.6142	2.8564
		Lim	0.5882	0.22877	0.073	-0.0328	1.2093
	Lim	AA	0.3529	0.22877	0.560	-0.2681	0.9740
		Nona	1.6471*	0.22877	<0.001	0.3248	1.6752
		HA	-0.5882	0.22877	0.073	-1.2093	0.0382

AA – Amyl-acetate, Nona – nonanal, HA – 2-hydroxactephenone, Lim – Limonene

The error term is Mean Square (Error) = 0.526

*The mean difference is significant at the 0.05 level.
